# Cartilage Protective and Immunomodulatory Features of Osteoarthritis Synovial Fluid-Treated Adipose-Derived Mesenchymal Stem Cells Secreted Factors and Extracellular Vesicles-Embedded miRNAs

**DOI:** 10.3390/cells10051072

**Published:** 2021-04-30

**Authors:** Enrico Ragni, Alessandra Colombini, Marco Viganò, Francesca Libonati, Carlotta Perucca Orfei, Luigi Zagra, Laura de Girolamo

**Affiliations:** 1 Laboratorio di Biotecnologie Applicate all’Ortopedia, IRCCS Istituto Ortopedico Galeazzi, I-20161 Milano, Italy; enrico.ragni@grupposandonato.it (E.R.); alessandra.colombini@grupposandonato.it (A.C.); marco.vigano@grupposandonato.it (M.V.); francesca.libonati@grupposandonato.it (F.L.); carlotta.perucca@grupposandonato.it (C.P.O.); 2 Hip Department, IRCCS Istituto Ortopedico Galeazzi, I-20161 Milano, Italy; luigi.zagra@fastwebnet.it

**Keywords:** osteoarthritis, synovial fluid, ASCs, secretome, extracellular vesicles, miRNAs, cytokines, chondrocytes

## Abstract

Intra-articular administration of adipose-derived mesenchymal stem cells (ASCs), either in vitro expanded or within adipose tissue-based products obtained at point-of-care, has gained popularity as innovative regenerative medicine approach for osteoarthritis (OA) treatment. ASCs can stimulate tissue repair and immunomodulation through paracrine factors, both soluble and extracellular vesicles (EV) embedded, collectively defining the secretome. Interaction with the degenerative/inflamed environment is a crucial factor in understanding the finely tuned molecular message but, to date, the majority of reports have described ASC-secretome features in resting conditions or under chemical stimuli far from the in vivo environment of degenerated OA joints. In this report, the secretory profile of ASCs treated with native synovial fluid from OA patients was evaluated, sifting 200 soluble factors and 754 EV-embedded miRNAs. Fifty-eight factors and 223 EV-miRNAs were identified, and discussed in the frame of cartilage and immune cell homeostasis. Bioinformatics gave a molecular basis for M2 macrophage polarization, T cell proliferation inhibition and T reg expansion enhancement, as well as cartilage protection, further confirmed in an in vitro model of OA chondrocytes. Moreover, a strong influence on immune cell chemotaxis emerged. In conclusion, obtained molecular data support the regenerative and immunomodulatory properties of ASCs when interacting with osteoarthritic joint environment.

## 1. Introduction

Osteoarthritis (OA) is a chronic degenerative joint disease that preferentially affects knees, hands, hips and the spine [1]. OA progression leads to synovitis, cartilage degradation and subchondral bone remodeling, joint capsule hypertrophy, and formation of osteophytes [2]. Moreover, OA is characterized by inflammation at different levels, with the direct contribution of fibroblast-like synoviocytes, chondrocytes and, mainly, immune cells such as macrophages and T cells [3]. The polarization of macrophages and the failure of synovial macrophages to transform from M1 to M2 subtypes has an effect on OA progression and the degree of M1/M2 imbalance is often associated with disease severity. Synovial macrophage activation to the M1 phenotype leads to the production of pro-inflammatory cytokines, including IL-12, IL-1β and TNFα, while M2 cytokine such as IL-1α is reduced, which worsens the osteoarthritic joints [4]. The overproduction of cytokines from the inflamed synovium can trigger the degradation of cartilage through the production of other pro-inflammatory cytokines, matrix metalloproteinases (MMPs), and aggrecanases [4]. Furthermore, both Th1 cells infiltrate the synovia and their pro-inflammatory mediators get predominant with respect to Th2 presence in most of OA patients [5]. Moreover, regulatory T lymphocytes (T reg) are found in both synovia and synovial fluid (SF) [6]. T reg presence in the joints is an attempt of the immune system to control the inflammatory responses, as they modulate the secretion of anti-inflammatory cytokines and the expression of receptors for cytokines [7]. In fact, T reg from OA patients were reported to produce anti-inflammatory IL-10 in the synovium [8]. As a consequence, a decrease in T reg response is supposed to be involved in the pathogenesis of OA. Therefore, due to its multifaceted nature, a number of controversies and challenges exist for the treatment of OA to avoid surgery that should be reserved for patients that have not responded appropriately to less invasive methods.

Standard conservative OA management is often limited to symptomatic treatments by, among others, steroids, nonsteroidal anti-inflammatory drugs and joint injections of hyaluronic acid [9]. Due to the limited efficacy of these approaches, in the last few years, regenerative medicine treatments prepared at the point-of-care have become increasingly popular. In particular, injection of plasma fraction enriched in platelets, bone marrow aspirate concentrate and processed adipose tissue, either in the form of stromal vascular fraction (SVF, obtained by enzymatic or mechanical digestion) or microfragmented (MFAT) by means of ultrasounds, pressure, shear and mechanical forces, are the most frequently used [9]. To date, although few clinical studies have been released for the use of SVF, preliminary results have demonstrated improved clinical outcomes, pain scores and functional status [10,11]. The rationale behind these adipose-based treatments is the presence of resident progenitor and stem cells (adipose-derived mesenchymal stem cells, ASCs) with regenerative and immunomodulatory features [12] within the adipose tissue. Consistently, two clinical trials using GMP-grade expanded ASCs have reported decreased cartilage defects and improvement in pain [13,14].

ASCs pro-regenerative effects might be ascribed to several features. Together with their capacity to differentiate towards the chondrogenic lineage, in a preclinical animal model of OA, ASCs were shown to migrate in the direction of the synovial membrane and of the meniscus following intra-articular delivery [15]. Furthermore, ASCs release anabolic, anti-fibrotic, and anti-apoptotic growth factors within the OA microenvironment, explaining the reduced degenerative responses [15,16,17]. Eventually, ASCs, again through secreted factors, could act as modulators of the immune response by both inducing macrophages to differentiate into anti-inflammatory regulatory M2 cells and reducing T cell proliferation and Th1/Th17 number [18].

Despite these pivotal data, the exact molecular mechanisms behind ASC functions in the OA environment are poorly deciphered. In fact, the vast majority of in vitro studies aimed at fingerprinting the ASC secretory portfolio and its molecular effects, although of pivotal importance, have been performed in standard culturing conditions or after different inflammatory priming strategies that were not resembling physiological conditions [19]. In fact, most of the OA model was created by single cytokine supplementation in the ng/mL range in spite of several factors in the pg/mL range that are known to characterize OA-SF [20]. To date, only a few reports have described the ASC response after direct interaction with OA-SF, confirming their ability to inhibit T cell proliferation and promote T reg expansion, induce macrophage polarization into the M2-like phenotype and reduce monocyte differentiation into mature dendritic cells [21,22,23,24]. By preliminary analyses on a reduced number of cytokines, these effects were postulated to rely on specific ASC secreted factors (secretome) in the presence of OA-SF. Of note, no data describing the effect of OA-SF-treated ASCs and their secretome on OA chondrocytes are available.

To obtain a more complete molecular picture, in this work the secretome released by ASCs after 48 h incubation with OA-SF have been sifted for a panel of 200 cytokines, chemokines and growth factors, as well as for a panel of 754 miRNAs embedded in secreted extracellular vesicles (EVs). Data have been analyzed by bioinformatic tools in the frame of joint tissues and cells, such as chondrocytes, macrophages, T reg and T cells, typically altered in an osteoarthritic condition. Predicted effects on cartilage have been validated in an in vitro model of inflamed OA chondrocytes. Overall, results support with molecular data the observed regenerative and anti-inflammatory effects in both pre-clinical and clinical settings for OA treatment.

## 2. Materials and Methods

### 2.1. Ethics Statement

The research was performed at IRCCS Istituto Ortopedico Galeazzi. Institutional Review Board approval (San Raffaele Hospital Ethics Committee approval on date 16 December 2020, registered under number 214/int/2020) was granted before the beginning of the study. Samples were collected after the procurement of patient informed consent and following the 1964 Helsinki declaration and its later amendments.

### 2.2. OA-SF Collection

SF was collected from six OA (Kellgren and Lawrence III–IV grade) patients who underwent total hip arthroplasty. The mean patient age at the time of surgery was 68 years (range 52–87 years). Subjects consisted of 3 males and 3 females. The OA-SF was collected by puncture before arthroplasty. OA-SF were centrifuged at 16,000× *g* for 10 min to remove debris, and the supernatants were immediately aliquoted and stored at −80 °C.

### 2.3. SF Cytokine Quantification

For each OA-SF, 600 μL were supplemented with 60 μL of 20 mg/mL Hyaluronidase (Sigma-Aldrich, Milan, Italy) and 3 μL of Protease Inhibitor Cocktail (abcam, Cambridge, MA, USA). Incubation was performed for 30 min at 37 °C. Undiluted digested OA-SF was used for cytokine (IL-1α, IL-1β, IL-2, IL-4, IL-6, IL-8, IL-10, IL-12, IL-17A, TNFα and GM-CSF) detection with the Multi Analyte Elisarray Kit (Qiagen, Hilden, Germany), following manufacturer’s protocol. Concentrations were determined by comparison with standard samples.

### 2.4. ASCs Isolation and Culture

Adipose waste material from three female donors (54 ± 8 years old) undergoing liposuction was processed as previously reported [25]. After 30 min digestion at 37 °C with type I collagenase (Worthington Biochemical Co., Lakewood, NJ, USA), tissues were filtered (100 μM cell strainer), centrifuged at 1000× *g* (5 min, RT) and pellets suspended in DMEM + 10% FBS (CM) before seeding at 5 × 10^3^ cells/cm^2^ (37 °C, 5% CO_2_, 95% humidity). ASCs were selected by plastic adherence and cultured until passage 5 for analyses.

### 2.5. ASCs Exposure to OA-SF

When indicated, ASCs at 90% confluence were washed twice with PBS and exposed to culture medium supplemented with 50% pooled OA-SF for 48 h. OA-SF pool was obtained adding the same volume of each OA-SF previously collected.

### 2.6. Cell Count and Viability

ASC number and viability were assayed with a NucleoCounter^®^ NC-3000™ Advanced Image Cytometer (ChemoMetec, Allerod, Denmark), following the manufacturer’s instructions.

### 2.7. ASCs Characterization by Flow Cytometry

ASCs, with and without 50% OA-SF, were analyzed by flow cytometry with a CytoFLEX flow cytometer (Beckman Coulter, Fullerton, CA, USA), collecting at least 50,000 events. Antibodies: anti-CD90-FITC (REA897, Miltenyi Biotec, Bergisch Gladbach, Germany), CD73-PE (REA804, Miltenyi), CD105-PerCP-Cy5.5 (43A3, BioLegend, San Diego, CA, USA), CD44-PE-Vio770 (REA690, Miltenyi), CD34-FITC (AC136, Miltenyi), CD271-PE (REA844, Miltenyi), CD31-PerCP-Vio700 (REA730, Miltenyi) and CD45-PE-Vio770 (REA747, Miltenyi). Aggregates were removed from gating events on a FSC-H and FSC-A plot.

### 2.8. Secretome Collection

After incubation with OA-SF, ASCs were extensively washed with PBS and fresh DMEM (12 mL per T175 cell culture flask) without FBS added. After 48 h at 37 °C, secretome was collected and centrifuged at 4 °C for 15 min at 1000× *g* and 2000× *g* and twice at 4000× *g* to remove broken cells and debris. Secretome was stored at −80 °C until further use.

### 2.9. ELISA Assay

Quantibody^®^ Human Cytokine Array 4000 Kit (https://www.raybiotech.com/quantibody-human-cytokine-array-4000/, accessed on April 2021) was used to determine the concentration of 200 soluble inflammatory and growth factors, chemokines, receptors and cytokines in the serially centrifuged secretome, according to the manufacturers’ instructions (RayBiotech, Norcross, GA, USA). Appropriate dilutions were made to have absorbance readings within the standard curve values. Only factors detected above a single assay threshold in all samples were considered. The absolute amount of each factor was obtained by multiplying the pg/mL concentration per the total volume of secretome, and eventually divided per million cells to obtain a pg or ng/10^6^ cell ratio. Mean values ± SD are presented.

### 2.10. Construction of Protein–Protein Interaction Networks

The online tool STRING (http://www.string-db.org, accessed on April 2021) [26] was used to build interactome maps of ELISA-identified proteins (database v11, data accessed: January 2021) with the following properties: (i) organism, Homo sapiens; (ii) meaning of network edges, condifence; (iii) active interaction sources, experiments and databases; (iv) minimum required interaction scores, medium confidence (0.400).

### 2.11. EV Detection by Nanoparticle Tracking Analysis (NTA)

EVs in the serially centrifuged secretome (1:6 diluted in PBS) were visualized by the NanoSight LM10-HS system (NanoSight Ltd., Amesbury, UK). Five recordings of 60 s were performed for each sample. Collected data were analyzed by the NTA software, providing concentration measurements and high-resolution particle size distribution profiles. The number of EVs per million ASCs was eventually calculated.

### 2.12. EV Characterization by Flow Cytometry

Forty-five μL of serially centrifuged secretome were supplemented with 5 μL of 10 μM CFSE (1 μM final) and incubation performed in the dark at 37 °C for 1 h. After addition of 50 μL PBS, one fifth of the labeled secretome was stored at 4 °C, whereas the rest was divided into 4 aliquots and each (20 μL) stained for 30 min at 4 °C in the dark with 1 μL of the following APC-conjugated Ab: anti-CD9 (312107, BioLegend), CD63 (353007, BioLegend), CD81 (349509, BioLegend) and CD73 (344005, BioLegend). Antibodies were used individually. After incubation, 80 μL PBS were added to each sample and events collection was performed with a CytoFLEX flow cytometer at 10 μL/min flow rate. Flow cytometer was set with reference Megamix-Plus SSC beads (Biocytex, Marseille, France) composed of FITC fluorescent spheres (160 nm, 200 nm, 240 nm, and 500 nm). FITC threshold was set at 500 to include 160 nm beads and some smaller debris in the FITC/CFSE channel.

### 2.13. EVs Isolation

Five millilitres of serially centrifuged secretome was 1:2 diluted with PBS and centrifuged at 100,000× *g* for 9 h at 4 °C in a 70Ti rotor (Beckman Coulter, Fullerton, CA, USA), and EV pellets processed for electron microscopy or miRNA expression.

### 2.14. Transmission Electron Microscopy (TEM)

After EV pellet suspension in PBS (100 μL per initial 5 mL secretome), 5 μL were absorbed for 10 min at RT on formvar carbon-coated grids. Filter paper was used to blot drops. Two percent uranyl acetate aqueous suspension was used for 10 min to perform negative staining. Excess was removed by filter paper. The grid was dried at RT. Samples were examined with a TALOS L120C transmission electron microscope (Thermo Fisher Scientific, Waltham, MA, USA) at 120 kV.

### 2.15. EV-Embedded miRNAs Expression

Trizol was used to dissolve EV pellets, followed by miRNeasy Kit and RNeasy CleanUp Kit to isolate RNA enriched in small molecules < 200 nt (Qiagen). Before each RNA extraction, 6 pg of a nonhuman synthetic miRNA spike-in (Arabidopsis thaliana ath-miR-159a) were added to monitor the technical variability during the whole detection procedure. ath-miR-159a was further used to equalize A and B panels of the OpenArray^®^ platform (Thermo Fisher Scientific). cDNAs were obtained by standard reverse transcription, with preamplification performed with A and B independent kits, followed by real-time RT-PCR analysis with the QuantStudio™ 12 K Flex OpenArray^®^ Platform (QS12KFlex), as previously described [27]. The Expression Suite Software (Thermo Fisher Scientific) processed miRNA expression data from A and B miRNA panels, covering 754 human miRNA sequences from the Sanger miRBase v21. C_RT_ values ≥ 28 were considered as unumplified samples, as per the manufacturer’s instructions. Normalization was performed using the global mean strategy [28], obtained considering only miRNAs positively detected in all samples. miRNA values are shown as normalized C_RT_, and mean values ± SD are presented.

### 2.16. EV-miRNAs Target the Identification and Functional Analysis

miRTarBase (http://mirtarbase.cuhk.edu.cn/php/index.php, accessed on April 2021) [29] was used to annotate the miRNA targets, selecting experimental strong evidence (reporter assay, Western blot and qPCR) as a validation method in place of solely bioinformatics prediction. All experimentally validated strong miRNA–mRNA interactions were submitted to GOrilla web interface (http://cbl-gorilla.cs.technion.ac.il/, accessed on April 2021) [30] to search for enriched Gene Ontology (GO) terms that appear densely at the top of a ranked list of genes (unbiased, against whole genome) or, after filtering againsts first quartile OA-cartilage or OA-sinovia infiltrative macrophages for shared candidates, for enriched GO terms in a target list of genes compared to a background list of genes (against the same OA cartilage or macrophage first quartile gene expression datasets), under default settings except for *p*-value threshold run as specified in the Results. Eventually, filtered interactions were also submitted to the PANTHER web interface (http://www.pantherdb.org/, accessed on April 2021) [31] to identify genes belonging to the same functional classifications, following default settings.

### 2.17. Hierarchical Clustering and Principal Component Analysis (PCA)

Heat map and PCA plots were generated, scoring factors or miRNAs, with ClustVis package (https://biit.cs.ut.ee/clustvis/, accessed on April 2021) [32]. Data pre-processing: no transformation, no centering, no scaling applied to raws and SVD with imputation for PCA method. Heat map clustering options: both rows and columns were clustered using correlation distance and average linkage. Ln(x) [(pg) factors per 10^6^ cells] or miRNA C_RT_ after normalization values were used.

### 2.18. Effect of Secretome from ASCs Treated with OA-SF on Inflamed Chondrocytes

Chondrocytes were obtained from 3 OA (Kellgren Lawrence III–IV grade) patients undergoing total hip arthroplasty [33]. The cartilage was harvested from non-weight bearing superficial areas of femoral head/neck and digested with 0.15% *w/v* type II collagenase (Worthington Biochemical, Lakewood, NJ, USA) at 37 °C for 22 h. Then, chondrocytes were isolated for plastic adherence and cultured in DMEM + 10% FBS until passage 3. For secretome functional analysis, when at 90% confluence, either control medium or control medium + 25 pg/mL IL-1β were added and maintained for 3 days. Afterwards, 3 conditions were run: control (DMEM + 10% FBS), 25 pg/mL IL-1β in control medium, or 25 pg/mL IL-1β in SF-treated ASCs secretome (+10% FBS) obtained pooling secretomes from the 3 ASCs under study. Ultracentrifuged FBS was used to avoid serum EVs interference. After 4 days, chondrocytes were counted and stained in the dark at 4 °C for 30 min with anti-VCAM1-APC (HA58, Miltenyi) and ICAM1-PE (REA269, Miltenyi). Unstained cells were used as negative control. At least 50,000 events were acquired with a CytoFLEX flow cyotometer (Beckman Coulter). Data are presented as mean ± SD of positive cells % for VCAM1 and Mean Fluorescence Intensity (MFI) ratio of treated vs. non-inflamed chondrocytes set as 1, after subtraction of MFI values of unstained counterparts, for ICAM1.

### 2.19. Comparison of OA-SF Treated vs. Untreted ASCs Secretome

Soluble factors and EV-miRNA expression data in control medium were retrieved from a previous publication form our group [33]. Donors, ASCs passage, culturing conditions (except OA-SF treatment) and technical platforms for ELISA and qRT-PCR were identical to those used in this study. For miRNA comparison, miR-16-5p/23a-3p/103a-3p/22-5p/26a-5p/29a-5p were selected as reference genes according to previous publications from our group [34,35,36,37].

### 2.20. Statistical Analysis

For the secretome effect on inflamed chondrocytes, statistical analysis was performed using the GraphPad Prism software (GraphPad, San Diego, CA, USA). Kolmogorov–Smirnov and Grubb’s tests were used. One sample t test was used to compare means vs. reference values set as 1, and *t* test of two means was used for other comparisons, with significance set at *p* value < 0.05. For the different bioinformatic tools used to sift factors or miRNA targets, the level of significance was set to a minimum *p*-value (*p*) of <0.05 or <10*^−^*^3^, depending on analyses and the algorithms’ parameters.

## 3. Results

### 3.1. OA-SF Characterization and ASCs Immunophenotype

A panel of inflammatory-related cytokines and chemokines was assayed in six OA-SF (Table 1). IL-6 and IL-8 resulted the most abundant factors, 865 ± 275 and 325 ± 74 pg/mL, respectively. The response of ASCs treated with OA-SF was monitored culturing cells in the absence or presence of 50% pooled OA-SF. OA-SF addition for 48 h induced cell proliferation (CM+OA-SF/CM ratio of 2.6 ± 0.1) without altering cell viability (90% ± 1 vs. 90% ± 3). ASC morphology became more irregular with several cytoplasmic protrusions (data not shown). Furthermore, ASCs were characterized by flow cytometry for both mesenchymal stem cell (MSCs) and hemato-endothelial markers (Supplementary Figure S1). In both conditions, ASCs were strongly positive for mesenchymal epitopes (CD44/73/90/105) and negative (<1%) for CD31 and CD45 epitopes, as well as for CD271. Similarly, weak ASC positivity for CD34 (7% ± 2 vs. 8% ± 2 for CM+OA-SF vs. CM) was not altered.

### 3.2. Soluble Factors Secreted from OA-SF-Treated ASCs

**Figure 1:**
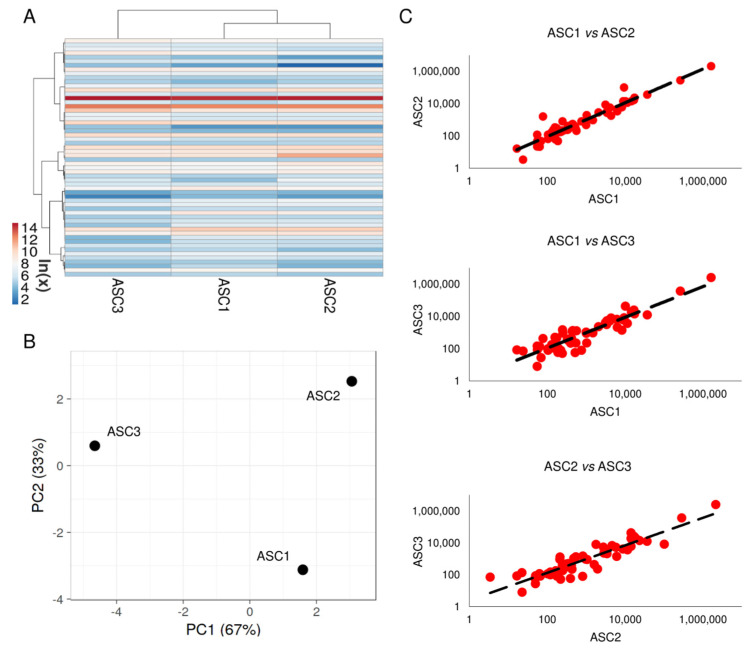
Comparison of secreted factor profiles between ASCs under study after OA-SF treatment. (**A**) Heat map of hierarchical clustering analysis of the ln(x) transformed pg/million ASC values of detected factors with sample clustering tree at the top. The color scale reflects the absolute expression levels: red shades = high expression levels and blue shades = lower expression levels. (**B**) Principal component analysis of the ln(x) transformed pg/million ASC values of detected factors. *X* and *Y* axis show principal component 1 and principal component 2 that explain 67% and 33% of the total variance. (**C**) Correlation of factors expression levels (expressed as pg/million ASC) between the three ASCs under study.

Two-hundred soluble factors, encompassing inflammatory, growth factors, chemokines, cytokines and receptors, were analyzed in ASC-conditioned medium after OA-SF treatment. Fifty-eight molecules were detected above the ELISA detection threshold and at varying levels of intensity in all donors (Supplementary Table S1). Hierarchical clustering showed higher similarity for ASC1 and 2 (Figure 1A), although in a context of conserved distance between ASCs, as highlighted by Principal Component Analysis and correlation analysis (mean R = 0.99 ± 0.01) (Figure 1B,C). Thus, average values for each detected factor were calculated in order to provide a guide to their levels (Supplementary Table S1). Considering one million cells, IGFBP4 and IGFBP6 were secreted with an amount higher than 100 ng (2110 ± 462 and 309 ± 59, respectively). Seven molecules were between 100 and 10 ng (SPP1 40 ± 54; FST 29 ± 14; PF4 (CXCL4) 23 ± 18; TIMP2 20 ± 5; SERPINE1 19 ± 5; PLAUR 18 ± 1 and TIMP1 16 ± 3). Fourteen factors were between 10 and 1 ng and 35 molecules resulted below 1 ng.

**Figure 2:**
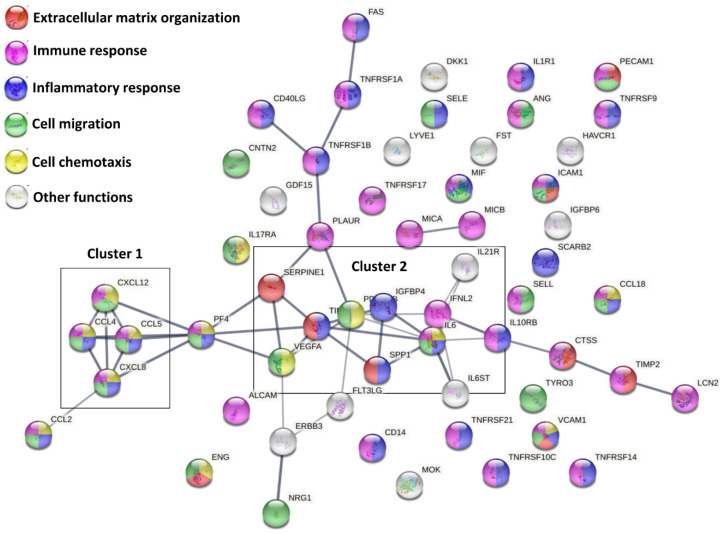
Functional association network for ELISA-detected secreted factors. Using the online tool STRING, protein–protein interaction levels for 58 proteins of the ASC secretome after OA-SF treatment were mined. Legend: factors involved in Extracellular matrix organization, red nodes; Immune response, purple nodes; Inflammatory response, blue nodes; Cell migration, green nodes; Cell chemotaxis, yellow nodes. Edges represent protein–protein associations, with thickness representing Edge confidence (medium: 0.400, high: 0.700, highest: 0.900). Empty nodes: proteins of unknown 3D structure; filled nodes: some 3D structure is known or predicted.

A functional protein association network analysis, based only on both experimental and database annotated interactions, allowed the definition of 2 main clusters, linked through PF4, and few lateral branches with less interconnections among molecules (Figure 2). The first cluster was tighter and characterized by a high degree of interactions between members (CCL4, CCL5, CXCL8 and CXCL12), and linked to CCL2. The second cluster was more heterogeneous and more branched. A deeper analysis (Figure 2 and Supplementary Table S2) of Biological Processes showed that, overall, 9 factors are involved with Extracellular matrix organization (GO:0030198, red nodes in Figure 2), 34 in Immune response (GO:0006955, purple nodes), 26 in Inflammatory response (GO:0006954, blue nodes), 22 in Cell migration (GO:0030198, green nodes) and 13 in Cell chemotaxis (GO:0060326, yellow nodes). The strong impact on cell locomotion was also present refining the search for leukocytes, with 16 factors involved in Leukocyte migration (GO:0050900) and 10 in Leukocyte chemotaxis (GO:0030595). A further narrowing for immune cells directly involved in OA, such as T cells and monocytes/macrophages, consistently evidenced GO terms related to locomotion as T cell migration (GO:0072678, 3 players) and Monocyte chemotaxis (GO:0002548, 5 players), including Macrophage chemotaxis (GO:0048246, 2 players). In particular, positive regulation of locomotion was defined for both T cell (GO:2000406, 2 players) and monocytes (GO:0090026, 3 players). Intriguingly, T cell extravasation was also identified (GO:0072683, 2 players). The overall influence on locomotion also defined GO terms related to other immune cells, such as Granulocyte migration (GO:0097530, 8 players) and chemotaxis (GO:0071621, 7 players), including Neutrophil chemotaxis (GO:0030593, 5 players) and migration (GO:1990266, 6 players), both positive (GO:0090023 and GO:1902624, 2 and 3 players). Regarding inflammatory/immune response, leukocytes resulted in the most involved cell type, with 14 factors defining Leukocyte mediated immunity (GO:0002443), mostly relying on Neutrophil-mediated immunity (GO:0002446, 10 players) and T cell-mediated immunity (GO:0002456, 3 players). This is also reflected by the 11 factors involved in the Regulation of cytokine production (GO:0001817). Moreover, TNFs resulted in the preferential target of secreted factors, being the GO term Cellular response to Tumor necrosis factor (GO:0071356) composed of 16 players. Consistently, 7 molecules resulted involved in signaling (GO:0005031), including 2 TNFα receptors (GO:0043120). Concerning other cytokines related to OA phenotype, also IL-1β, IL-17 and IL-21 receptors (IL-1R1, GO:0004908; IL-17R, GO:0030368; and IL-21R, GO:0001532) were identified. Accordingly, 7 factors were involved in Cellular response to Interleukin-1 (GO:0071347). Of note, 6 factors defining Cellular response to Interferon-gamma (GO:0071346) and 3 shaping Cellular response to Interleukin-6 (GO:0071354), both cytokines involved in OA, also emerged. For this last GO term, both IL6ST, encoding for Interleukin-6 receptor subunit beta (GO:0004896) and IL6 itself (GO:0005125) were present, with IL6ST 20 times more abundant. Eventually, among binding proteins/receptors, IGFI/II binding proteins (GO:0031994/5, 2 factors each) resulted the most abundant factors.

### 3.3. Characterization of Extracellular Vesicles Released from OA-SF-Treated ASCs

**Figure 3:**
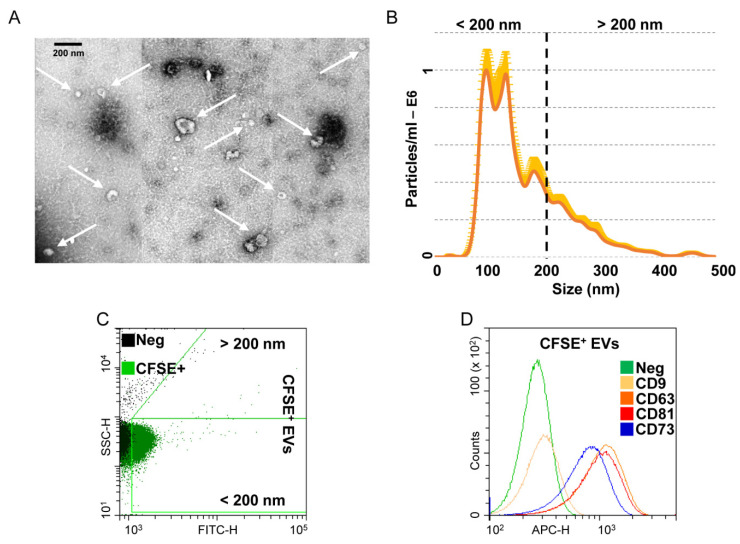
Characterization of EVs from OA-SF treated ASCs. (**A**) Transmission electron micrographs of EVs showing characteristic cup-shaped morphology. (**B**) Mean particle size analysis from NTA data. (**C**,**D**) EVs flow cytometry analysis. EVs were CFSE stained to allow identification and gating of vesicles in the FITC channel. After gating, CFSE+ EVs showed positive staining for extracellular vesicle defining molecules CD63 and CD81 and MSC marker CD73. CD9, another EV postulated marker, was barely detectable. Representative cytograms are presented.

OA-SF-treated ASCs released 1.8 × 10^9^ ± 0.3 EVs per 10^6^ cells in 48 h. At TEM, EVs exhibited the characteristic cup-shaped morphology (Figure 3A). NTA analysis confirmed the nanometer-scale expected EVs size range, mean 161 ± 3 nm and mode 103 ± 12 nm, with enrichment in small particles (75% ± 3 below 200 nm), possibly exosomes (Figure 3B). After flow cytometer calibration with FITC-fluorescent nanobeads of predetermined size (160 to 500 nm, Supplementary Figure S2), detected CFSE-labelled EVs size confirmed NTA dimensional range (84% ± 1 below 200 nm) (Figure 3C). EVs resulted strongly positive for extracellular vesicles markers CD63 (92% ± 1) and CD81 (91% ± 1), and for MSC lineage marker CD73 (83% ± 1). CD9, another postulated EVs marker, was weakly expressed (6% ± 1) (Figure 3D).

### 3.4. EVs Associated miRNAs

**Figure 4:**
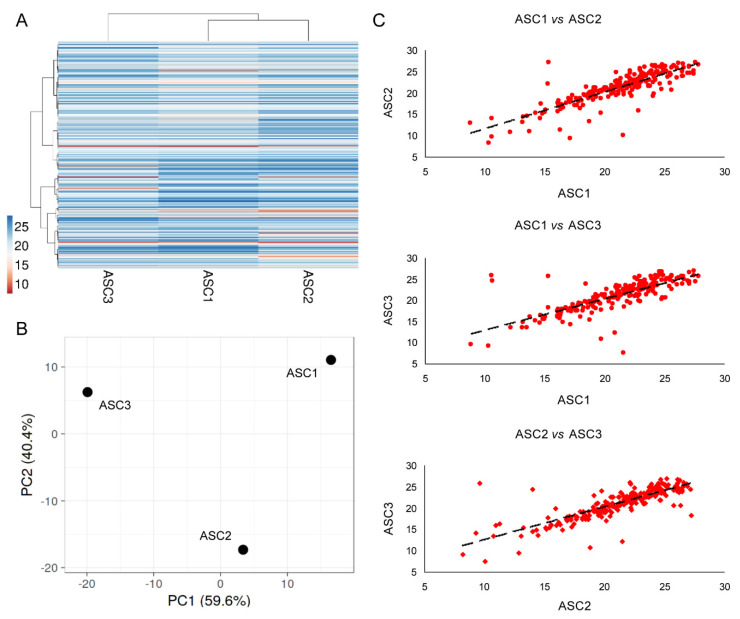
Comparison of EV-miRNA expression profiles between ASCs under study after OA-SF treatment. (**A**) Heat map of hierarchical clustering analysis of the normalized C_RT_ values of detected miRNAs with sample clustering tree at the top. The color scale reflects the absolute expression levels: red shades = high expression levels (low C_RT_ values) and blue shades = lower expression levels (high C_RT_ values). (**B**) Principal component analysis of the normalized C_RT_ values of detected miRNAs. *X* and *Y* axis show principal component 1 and principal component 2 that explain 59.6% and 40.4% of the total variance. (**C**) Correlation of miRNA expression levels (normalized C_RT_) between the three ASC-EVs under study.

Two hundred and twenty-three EV-miRNAs were detected in all the three OA-SF-treated ASCs (Supplementary Table S3). As for secreted factors, ASC1 and 2 were more similar (Figure 4A), although again had conserved a distance between all the ASCs, as highlighted by Principal Component Analysis and correlation analysis (mean R = 0.81 ± 0.04) (Figure 4B,C). Thus, an average C_RT_ value for each EV-embedded miRNA was calculated in order to provide a guide to its level (Supplementary Table S3). Ninety-one per cent of miRNAs had a SD ≤ 2, and 97% ≤ 4.

To attribute a biological significance to EV-miRNAs, recent reports showed that even for most abundant ones there is around 1.3 molecule per MSC vesicle [38], and almost 100 EVs are necessary to transfer one copy of an abundant miRNA to a target cell/tissue [39]. Therefore, to get a solid picture influenced as little as possible by donor-dependent fluctuations, within the group having a SD ≤ 2 we focused our attention on the miRNAs falling in the first quartile of detection (Supplementary Table S4). Due to its high expression (second overall best with mean C_RT_ of 10.41) and being the first excluded by the SD cutoff (SD of 2.16), miR-518f-3p was also included. Collectively, 51 selected miRNAs covered the 88% of the whole genetic message (Supplementary Table S4). By mining only miRNA–mRNA interactions was defined by strong experimental evidence, 47 out of 51 miRNAs were reported to target from one to several genes (Supplementary Table S5). Notably, to date (April 2021), the 3 most abundant miRNAs (mir-520e-3p, miR-518f-3p and miR-523-3p) were not annotated in the database. Overall, 47 miRNAs univocally target 954 genes (Supplementary Table S6). An unbiased GO enrichment analysis against the whole genome, with a stringent *p*-value threshold (<10^−9^) due to the absence of a background gene expression dataset refining the search, was able to identify few terms related to protein kinase activity, namely BP regulation of cyclin-dependent protein kinase activity (*p* = 5.16 × 10**^−^**^10^, GO:1904029) and regulation of cyclin-dependent protein serine/threonine kinase activity (5.16 × 10**^−^**^10^, GO:0000079), MF cyclin-dependent protein serine/threonine kinase regulator activity (4.85 × 10**^−^**^12^, GO:0016538), and CC serine/threonine protein kinase complex (4.55 × 10**^−^**^10^, GO:1902554) and cyclin-dependent protein kinase holoenzyme complex (9.05 × 10**^−^**^10^, GO:0000307). This was further supported by 469 genes involved with binding (GO:0005488), 288 with catalytic activity (GO:0003824) and 249 with regulator molecular function (GO:0098772).

### 3.5. Target and Effect Prediction of EV-miRNAs on OA-Cartilage

To relate the effect of EV-miRNAs in the OA setting, the 954 genes were filtered through 2368 genes identified as most abundantly expressed in cartilage biopsies from OA patients (laying in the first quartile of expression out of 9474 total detected transcripts) [40]. After filtering, 270 genes emerged as shared (Supplementary Table S7). GO enrichment (*p* < 10^−9^) was performed by scoring identified genes vs. the first quartile of OA cartilage as background. Several BP terms emerged (Supplementary Table S8), suggesting that when a tissue and/or disease-focused search is conducted, the power and specificity of the outcomes increase. In particular, among the most significantly enriched GO terms, many are related with proliferation/migration (e.g., 5.88 × 10**^−^**^26^, GO:0042127, regulation of cell proliferation; 5.81 × 10**^−^**^22^, GO:2000145, regulation of cell motility; 3.03 × 10**^−^**^21^, GO:0030334, regulation of cell migration; 4.37 × 10**^−^**^21^, GO:0040012, regulation of locomotion), development/differentiation (e.g., 2.07 × 10**^−^**^25^, GO:0050793, regulation of developmental process, 2.28 × 10**^−^**^23^, GO:0030154, cell differentiation; 5.57 × 10**^−^**^22^, GO:0048869, cellular developmental process) and, with lower *p*-values, apoptosis/cell death (e.g., 1.89 × 10**^−^**^19^, GO:0010941, regulation of cell death; 2.17 × 10**^−^**^18^, GO:0042981, regulation of apoptotic process; 3.49 × 10**^−^**^18^, GO:0043067, regulation of programmed cell death). All these BP regulating the cell response and adaptation relies on several transcriptional regulation cascades ending with MF terms DNA-binding transcription activator activity, RNA polymerase II-specific (7.77 × 10**^−^**^10^, GO:0001228) and RNA polymerase II proximal promoter sequence-specific DNA binding (5.84 × 10**^−^**^13^, GO:0000978), strictly connected with CC term chromatin (7.85 × 10**^−^**^12^, GO:0000785).

Eventually, we focused our attention on miRNAs reported to be directly involved with OA-cartilage pathogenesis [41]. Fourteen protective and 6 degenerative miRNAs were identified (Table 2). Scoring EV-miRNA abundance, as a whole, protective miRNAs were 3.25 fold more present, due mainly to the detection of miR-193b3p/24-3p/92a-3p vs. the solely miR-21-5p in the top (>1% of the genetic weight) positions of the ranking. Notably, 3 miRNAs associated with overlapping roles in OA cartilage were present, with miR-125b-5p being the most abundant, and altogether showing a load comparable to the harmful ones (ratio of 0.89). Therefore, overall, cartilage protective signals far exceeded damaging inputs. Consistently, out of previously described 270 shared transcripts, several were annotated as involved in either cartilage/chondrocyte development or extracellular matrix organization (Supplementary Table S9). In particular, *SOX9*/*TGFBR2* are related with chondrocyte hypertrophy, *CTNNB1*/*SNAI2*/*SOX9*/*TGFBR1* with negative regulation of chondrocyte/cartilage development and *CAPNS1*/*CD44*/*FURIN*/*MMP2*/*MMP3*/*MMP14*/*TIMP2* with extracellular matrix disassembly.

### 3.6. Target and Effect Prediction of EV-miRNAs on OA-Macrophages

To get a more exhaustive picture of EV-miRNAs in OA setting, the 954 genes were also filtered against the first quartile of expression (2498 transcripts) of infiltrated synovial macrophages from OA patients (total of 9991 transcripts) [42], and 264 shared candidates were identified. One hundred and twenty-seven genes were shared between the OA cartilage and synovial macrophages, suggesting a partially divergent response (Supplementary Table S10). Thus, the 264 genes were searched (*p* < 10^−9^) against the OA synovial-macrophages first quartile mRNAs as background to identify enriched GO terms. Again, several BP emerged, with partial overlap in respect to cartilage (Supplementary Table S11). Among the most significant, many GO terms are related with cellular processes (e.g., 5.7 × 10**^−^**^24^, GO:0048522, positive regulation of cellular process; 1.92 × 10**^−^**^22^, GO:0048518, positive regulation of biological process; 2.39 × 10**^−^**^20^, GO:0050793, regulation of developmental process), mainly metabolic (e.g., 1 × 10**^−^**^18^, GO:0031325, positive regulation of cellular metabolic process; 1.24 × 10**^−^**^18^, GO:0009893, positive regulation of metabolic process; 1.57 × 10**^−^**^18^, GO:0031323, regulation of cellular metabolic process) and apoptosis/cell death (e.g., 6.29 × 10**^−^**^19^, GO:0043067, regulation of programmed cell death; 2.58 × 10**^−^**^18^, GO:0042981, regulation of apoptotic process; 3.25 × 10**^−^**^18^, GO:0010941, regulation of cell death). With respect to cartilage, GO terms related with proliferation/migration, although among the most significant ones, showed lower *p*-values (e.g., 2.94 × 10**^−^**^17^, GO:0042127, regulation of cell proliferation; 2.34 × 10**^−^**^15^, GO:2000145, regulation of cell motility; 2.55 × 10**^−^**^15^, GO:0030334, regulation of cell migration; 1.63 × 10**^−^**^15^, GO:0040012, regulation of locomotion). Again, transcriptional regulation modulation underlies EV-miRNAs influence on OA-macrophages, as indicated by MF RNA polymerase II proximal promoter sequence-specific DNA binding (5.66 × 10**^−^**^13^, GO:0000978) and CC chromatin (7.53 × 10**^−^**^14^, GO:0000785) terms.

Eventually, we had a closer look to miRNAs recently published as orchestrating the macrophage M1 (pro-inflammatory) to M2 (anti-inflammatory) switch and phenotype [43]. Four miRNAs involved in M2, and 3 involved in M1 phenotype regulation were identified (Table 2). The presence of highly present miR-24-3p, responsible for M2 differentiation by blocking M1 activation, tips the balance towards a more pronounced influence on M2 phenotype, further supported by the overall 4.24 times higher abundance of M2-related miRNAs. Consistently, out of previously described 264 shared transcripts, 9 are annotated as involved in macrophage differentiation and 7 in macrophage activation (Supplementary Table S12). Intriguingly, as seen in OA-cartilage, extracellular matrix disassembly related *CAPNS1*/*CD44*/*FURIN*/*MMP2*/*MMP3*/*MMP14*/*TIMP2* transcripts emerged. Moreover, canonical M1 inflammatory cytokines laying in the first quartile of expression in OA-synovial macrophages mRNAs were targeted by EV-miRNAs (*TNFα*, *IL-1β* and *IL-6*). Overall, these data suggest a preponderance for M2 resolving macrophage polarization, and the presence of miR-16-5p, regulating the monocyte to macrophage developmental transition, with miR-34a-5p and miR-132-3p, participating in macrophage maturation, supported a wide influence of EV-miRNAs on monocyte/macrophage homeostasis.

### 3.7. Target and Effect Prediction of EV-miRNAs on T Cell and T Reg Proliferation

At present, transcriptomes from OA T cells and T reg are not available. Nevertheless, a recent in vitro study showed proliferation inhibition for T cells and promotion for T reg in presence of OA-SF treated ASCs-secretome [21]. First, we compared abundant EV-miRNAs with those reported to have an experimental effect on activation and immunoregulatory molecules in T cells [44]. Sifting first quartile EV-miRNAs, 9 players with dampening vs. 6 with boosting roles emerged (Table 2), with an overall ratio of 2.41 for T cell activation inhibitors. Notably, input to preserve homeostasis was mainly granted by miR-24-3p and miR-21-5p (>1% of the genetic weight) positions of the ranking. Furthermore, 5 of the identified miRNAs (miR-24-3p/21-5p/125b/5p/29a-3p/27a-3p) were described to reduce IFNγ expression, while only miR-19b-3p was able to promote its upregulation. Second, focusing the search on miRNAs was reported to have a specific influence on T reg activation/proliferation [45]: 2 resulted in inducing, 4 in blocking and 1 had an ambiguous role (Table 2). Intriguingly, overall, promoting miRNAs far exceeded by 2.84 fold those players reducing T reg expansion and activation. This was mainly due to the presence of miR-21-5p. Furthermore, miR-155-5p, crucial for T reg proliferation by inducing IL2 receptors, was also detected in EV miRNAs, although at low levels.

### 3.8. Validation of OA-SF Stimulated ASCs-Secretome on Inflamed Chondrocytes

**Figure 5:**
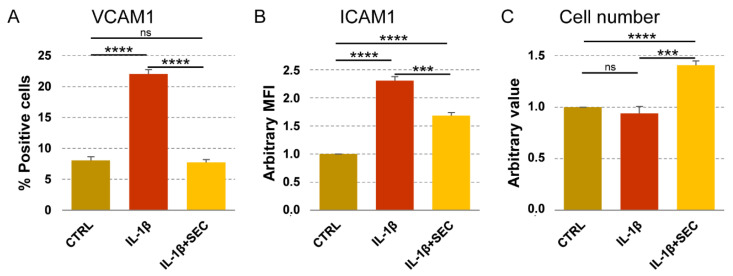
Secretome effects on inflamed chondrocytes. (**A**,**B**) Chondrocytes (CTRL) were treated with inflammatory cytokine without (IL-1β) and with secretome (IL-1β + SEC). VCAM1 and ICAM1 were detected by flow cytometry. Values on the *y*-axis show either the percentage of VCAM1-positive cells or the arbitrary amount (defined as Mean Fluorescence Intensity, being 100% chondrocytes positive in all conditions, CTRL set as 1) of ICAM1. *N* = 3, *** *p* ≤ 0.001, **** *p* ≤ 0.0001; ns, not significant. (**C**) Chondrocytes, as previously described, were counted and CTRL values set as 1. *N* = 3, *** *p* ≤ 0.001, **** *p* ≤  0.0001; ns, not significant.

To integrate available data of OA-SF-treated ASCs, secretome effects on OA-related immune cells such as macrophages, T cell and T reg [21], we further tested its activity on chondrocytes cultured under OA-mimicking stimuli [46]. Flow cytometry was used to test VCAM1 and ICAM1 positivity, their responsiveness being to inflammation and environmental metabolic alterations reported in chondrocytes [47,48,49]. IL-1β treatment significantly (*p* < 0.0001) upregulated VCAM1 expression, from 8% ± 1 to 22% ± 1 (Figure 5A). The secretome was able, even in presence of the inflammatory stimulus, to completely abolish the increase of VCAM1+ chondrocytes (*p* = ns vs. CTRL; *p* < 0.0001 vs. IL-1β). Inflammation also increased ICAM1 levels (Figure 5B) that, in all conditions being expressed in 100% of cells, were calculated as Mean Fluorescence Intensity (MFI), arbitrarily setting CTRL values as 1. IL-1β upregulated MFI values of a 2.3 ± 0.1 factor (*p* < 0.0001), but in the presence of the secretome, the ICAM1 levels were reduced to 1.7 ± 0.1 (*p* < 0.0001 vs. CTRL; *p* = 0.0002 vs. IL-1β). Notably, 4 days of secretome treatment was able to increase chondrocyte proliferation of a 41% ± 4 factor (*p* < 0.0001), while IL-1β alone did not affect cell number (Figure 5C). Overall, data supported secretome bioinformatics-predicted effects on inflamed chondrocyte homeostasis restoration and proliferation induction.

### 3.9. OA-SF Effect on ASC-Secreted Factors and EV-miRNAs

**Figure 6:**
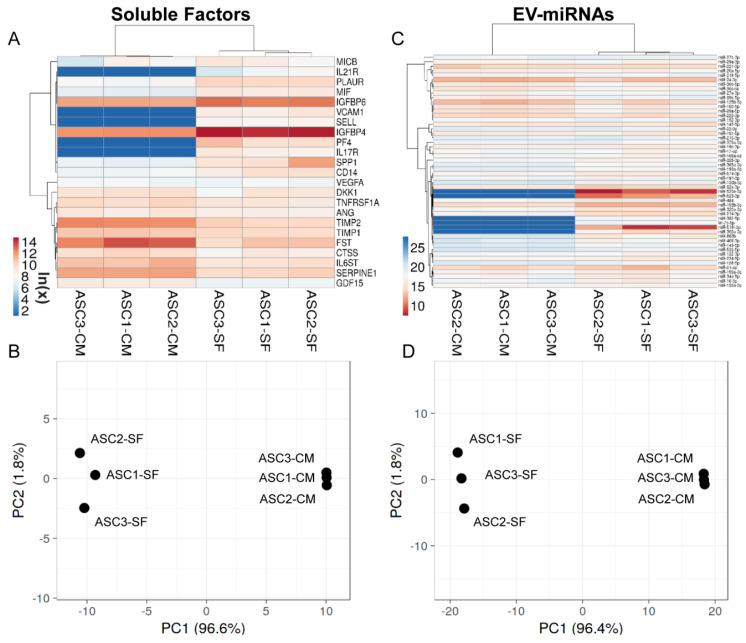
Comparison of abundant OA-SF-treated ASC (ASC-SF) soluble factors (>1 ng per million ASCs) and EV-miRNAs (in the first quartile of expression) with untreated counterparts in complete medium (ASC-CM). (**A**,**C**) Heat map of hierarchical clustering analysis of the ln(x) transformed pg/million ASC values of detected factors and normalized C_RT_ values of detected miRNAs with sample clustering tree at the top. The color scale reflects the absolute expression levels: red shades = high expression levels and blue shades = lower expression levels. (**B**,**D**) Principal component analysis of the ln(x) transformed pg/million ASC values of detected factors and normalized C_RT_ values of miRNAs. *X* and *Y* axis show principal component 1 and principal component 2 that explain 96.6% and 1.8% of the total variance (factors) and 96.4% and 1.8% of the total variance (miRNAs).

The levels of the most relevant factors (mean > 1 ng per million cells) and EV-miRNAs (mean laying in the first quartile of expression) have been compared to values previously published from our group reporting the secretome of the same cell isolates cultured in standard growth medium and analysed with identical ELISA and qRT-PCR platforms [33]. Regarding soluble factors, synovial fluid was able to deeply influence their levels (Supplementary Table S13), as highlighted by PCA and hierarchical clustering that showed a sharp dichotomy between groups (Figure 6A,B). In particular, out of 23 scored molecules, 5 gained (PF4, VCAM1, IL17R, SELL and IL21R), 3 increased (ratio > 2, *p*-value < 0.05; IGFBP4/6 and PLAUR) and 7 were reduced (ratio < 0.5, *p*-value < 0.05; FST, TIMP2, SERPINE1, IL6ST, TNFRS1A, CTSS and GCF15). Sifting previously described Biological Processes, gained and increased factors emerged as being involved with Immune responses (GO:0006955, SELL, VCAM1, PF4 and PLAUR), Inflammatory response (GO:0006954, VCAM1, PF4 and IGFBP4) and Cell chemotaxis (GO:0060326, IL17R, VCAM1 and PF4), with the term Locomotion (GO:0040011) defined by 5 terms out of 8 (IL17R, IL21R, VCAM1, PF4 and PLAUR). Notably, upregulation of both IGFBPs framed Insulin-like growth factor I/II binding BPs (GO:0031994/0031995). On the contrary, reduced factors described a more heterogeneous scenario, not able to sharply define any of the abovementioned BPs. Nevertheless, related BPs were Extracellular matrix organization (GO:0030198, CTSS, SERPINE1 and TIMP2) and disassembly (GO:0022617, CTSS and TIMP2), Immune system process (GO:0002376, TNFRSF1A, FST, TIMP2 and CTSS) and Positive regulation of inflammatory response (GO:0050729, TNFRSF1A, SERPINE1 and IL6ST).

Consistently, PCA and hierarchical clustering of abundant EV-miRNAs highlighted sharp separation between ASCs in the presence and absence of OA-SF (Figure 6C,D). With respect to soluble factors, the percentage of significantly (ratio < 0.5 or >2, *p*-value < 0.05) differentially expressed miRNAs was lower, with 6 gained (miR-520e-3p/518f-3p/523-3p/302a-3p/382-5p and let-7b-5p), 5 increased (miR-193b-3p/92a-3p/214-3p/484/130b-3p) and 6 decreased (miR-125b-5p/100-5p/99a-5p/30b-5p/30c-5p/99b-5p) out of 52 analysed molecules (Supplementary Table S14). Intriguingly, the 3 most expressed miRNAs (miR-520e-3p/518f-3p/523-3p) were not present in the secretome of untreated ASCs. By mining miRNA–mRNA interactions, gained/upregulated or downregulated miRNAs were reported to target 140 and 134 genes, respectively (Supplementary Table S15). Moreover, to avoid ambiguous results, shared genes between the two lists were removed, ending in 123 and 117 genes, respectively (Supplementary Table S15). Further filtering through abundantly expressed OA cartilage or macrophage genes (first quartile) allowed the definition of 47 and 37 genes for gained/upregulated miRNAs (cartilage or macrophages, respectively) and 21 and 26 genes for downregulated miRNAs (Supplementary Table S15). Analysing identified genes in the frame of Supplementary Tables S9 and S12 annotations, gained/upregulated miRNAs resulted in preferentially targeted molecules involved in chondrocyte/cartilage development and extracellular matrix organization (Supplementary Table S16), while a similar effect was not detected for downregulated miRNAs. Furthermore, macrophage-related categories were not massively targeted by both classes of miRNAs. Eventually, miRNAs directly involved in cartilage, macrophage, T cell and T reg homeostasis and reported in Table 2 were also studied (Table 3). Intriguingly, miR-193b-3p and miR-92a-3p, both exceeding the 1% EV-weight and reported to be chondro-protective, resulted in upregulation of a > 3.5 factor after OA-SF treatment. On the contrary, reduction of miR-125b-5p and upregulation of miR-214-3p, both exceeding 0.5%, might suggest a reduced ability to counteract T cell activation.

## 4. Discussion

In this report, both soluble factors and EV-embedded miRNAs, collectively being a crucial part of the secretome, have been characterized in ASCs treated with synovial fluid from OA patients. Our molecular data showed that several players have anti-inflammatory and pro-regenerative features, confirmed in vitro on OA chondrocytes, supporting the efficacy observed when ASCs-based treatments, either in the form of SVF/MFAT or culture-expanded, have been used in OA patients.

Out of 58 detected factors, 9 are directly connected with the GO term extracellular matrix (ECM) organization (Supplementary Table S2), with even more acting on ECM indirectly. Two MMP inhibitors (TIMP1 and 2) were amongst the most abundant factors (>10 ng per million ASCs) and this could exert a protective effect on OA cartilage. In fact, in animal models, TIMPs were shown to decrease in OA cartilage [50] and synovial fluid [51], leading to acceleration of OA progression [52]. Consistently, in the secretome of Wharton’s Jelly MSCs TIMP1/2 could support the prevention of cartilage degradation in both an in vivo [53] and a phase I/II study in humans [54]. The secretome protective effect on cartilage homeostasis could also rely on other molecules. Serpine1 (plasminogen activator inhibitor 1), as well as other Serpins, is upregulated in chondrocytes after cytokine stimulation [55] and in OA cartilage [56], and directly prevents both MMPs activity [57] and plasmin formation [58] by counteracting the urokinase-type and tissue-type plasminogen activators (uPA and tPA, respectively) [55], both elevated in OA cartilage [59]. Plasmin, the active form of plasminogen, can degrade the ECM by activating MMPs [60] and by cleaving structural components such as fibronectin, glycoproteins and proteoglycans [55]. Protection of proteoglycan erosion may also be exerted by follistatin, an activin receptor [61]. In addition, the two most abundant factors, IGFBP4 and 6, are positively linked with ECM homeostasis. IGFBPs regulate IGF1 (insuline-like growth factor 1) bioavailability and activity by reducing IGF1R-dependent sequestration and also protecting IGF1 from pericellular proteases [62]. This is of relevance since IGF1 was shown to stimulate ECM synthesis and cell proliferation in cartilage [63]. Moreover, IGFPBs are naturally located within the fibronectin network of the cartilage ECM [64], thus their supplementation with the secretome may increase cartilage IGF1 reservoirs, positively affecting its homeostasis and repair. Although not directly involved in ECM synthesis and architecture, other molecules may control its balance. TNFα, IL-1β, IL-17 and IL-21 receptors can reduce the presence of the respective cytokines that, by inducing and supporting inflammation, contribute to MMP expression and ECM degradation [65]. Of note, TNFα, IL-1β and IFNγ, among the most studied OA activators, were not detected in the secretome. Eventually, few molecules had an ambiguous or detrimental role on ECM. SPP1 (osteopontin), the third factor in the secretome, can specifically stimulate calcium pyrophosphate dihydrate (CPPD) crystal formation when incorporated in the pericellular matrix of osteoarthritic cartilage, inducing, in vitro, the release of catabolic cytokines and proteases from synovial cells and chondrocytes [66] and, clinically, more rapid progression of joint destruction [67]. Nevertheless, when osteopontin remains soluble at high levels and is not incorporated into ECM, exceeding the incorporation capacity of the matrix, it was suggested to inhibit CPPD crystal growth [68]. Another molecule with a possible dual role is PLAUR (uPA receptor), also present at high levels (>10 ng per million ASCs). At the plasma membrane level, PLAUR binds uPA triggering plasmin activation. However, uPA/PLAUR binding stimulates plasmin generation-independent events including chondrocyte proliferation [69]. Moreover, together with its degenerative features, plasmin may influence proliferation by activating growth factors including TGFβ, a molecule that plays a critical role in the development, growth, maintenance and repair of articular cartilage [70]. At last, CTSS (Cathepsin S) is involved in cartilage ECM degradation in vivo. Consistently, a collagen-induced arthritis mouse model showed that disease progression was diminished in Cathepsin S-deficient animals [71]. Overall, detected soluble factors have a preponderant protective effect on cartilage and ECM, although a more comprehensive picture might refine secretome and ASCs secretory significance.

Together with ECM, secreted factors defined several GO terms connected with cells of the immune system and their motility (Supplementary Table S2). In particular, 16 factors are involved in leukocyte migration, encompassing T cell, monocytes and granulocytes, particularly neutrophils. This is consistent with in vitro secretome reported chemo-attraction for several immune cells, further enhanced by inflammatory milieu [72,73]. Regarding OA-related joint immune cells, T cell and monocyte/macrophages, the majority of factors are within and connect with Cluster 1 (Figure 2) (CCL2/4/5 and CXCL12). Intriguingly, CCL2 with ICAM1 define the GO term T cell extravasation. Therefore, following soluble factor gradient and eventual migration, T cells and monocyte/macrophages may get close and interact with ASCs. Consistently, OA-SF treated ASC secretome, when directly interacting with cells of the immune system in a similar way to what may happen with proximity, was shown to have anti-inflammatory and immunomodulatory effects in vitro on macrophages and T lymphocytes, with T cell proliferation reduction and M2 macrophage switch enhancement alongside TNFα secretion mitigation [21]. This capacity is herein further supported by several soluble factors defining the GO terms, regulation of cytokine production, including TNFα, IL-1β and IL6 receptors, and cellular response to TNFα, IL-1β, IL6 and IFNγ. Thus, a scenario where ASCs, through secretion of soluble factors, call and accumulate at their proximity cells of the immune system in order to establish a microenvironment in which enriched locally acting factors such as soluble cytokine/chemokines and other regulatory molecules, such as EV-shuttled nucleic acids, amplify and coordinate a cross-talk able to reduce inflammation may be envisioned. This idea is supported by data showing that the secretome from MSCs in resting conditions or even pre-conditioned with single factors mimicking a multi-faceted scenario has reduced the effect with respect to secretome obtained after interaction with a more complex environment [74], herein simulated by natural factors present in tested OA-SF. In this frame, we are aware that the presence in OA-SF of immune cells, herein removed for long-term storage demand and able to directly interact with ASCs, might further enhance their response, especially if envisioned in an allogenic context, this scenario being the object of future investigations. Nevertheless, the immune privileged ASC features should allow for cell-to-cell cross-talk avoided by major immune reactions.

EV-miRNAs also supported the in vitro anti-inflammatory and protective effects on macrophages, T cell/T reg (as previously published, [21]) and inflamed chondrocytes. This would also reinforce the reduction in pain and inflammation, together with cartilage homeostasis improvement, observed in clinical trials [75]. miR-193b-3p, miR-24-3p and miR-92a-3p vs. miR-21-5p in the group with genetic weight > 1% shaped cartilage-protective signals (Table 2). miR-193b-3p, reduced in OA chondrocytes [76], has a putative binding site on the 3′-untranslated region of *MMP19*, which is increased in OA cartilage and synovium [77], and inhibits the production of nitric oxide [78], that is involved in cartilage degeneration [79]. Furthermore, miR-193b-3p overexpression increased histone H3 acetylation and transcription in the *COL2A1*, *AGGRECAN*, *COMP* and *SOX9* promoters [76]. Consistently, miR-193b overexpression strongly enhanced in vivo cartilage formation [76]. miR-24-3p, downregulated in OA cartilage reduces both metalloproteases secretion and chondrocyte senescence, positively regulating cartilage catabolism [80]. Accordingly, miR-24-3p was reported to promote chondrocyte proliferation (as observed in Figure 5C) and inhibit apoptosis [81]. miR-92a-3p, repressed in OA cartilage, enhances H3 acetylation and expression of *ACAN*, *COMP* and *COL2A1* promoters [82], and reduces aggrecan catabolism [83], thus protecting cartilage from proteolytic ECM destruction. Eventually, miR-21-5p, overexpressed in OA chondrocytes, attenuates the process of chondrogenesis by targeting growth differentiation factor 5 [84] that promotes chondrocyte differentiation [85]. Concerning macrophages, M2-related miRNAs in higher abundance is mainly due to miR-24-3p (Table 2). miR-24-3p overexpression significantly inhibits Mφ activation and M1 polarization with related cytokine expression, such as TNFα and IL6, in response to inflammatory stimuli, while increases M2 phenotype and associated markers (Arg1, CCL17, CCL22, CD163 and CD206) [86]. This is reinforced by EV-miRNA targeting of OA-synovial macrophage mRNAs encoding M1 inflammatory cytokines, such as TNFα, IL-1β and IL6, that, in combination with their soluble receptors, especially for TNFα, suggests a dual counteracting role against inflammatory mediators at both protein synthesis and availability levels. Of note, EV-miRNAs also target several OA-macrophage transcripts related to ECM disassembly, including *MMP2*/*MMP3*/*MMP14*, contributing to paracrine interactions between macrophages and chondrocytes. Thus, since the activation state and the M1/M2 ratio is highly associated with OA severity [87], EV-miRNA macrophage reprogramming from the M1 to M2 subtype, more than a decrease in the quantity of activated macrophages, might play a crucial role by skewing the inflammatory microenvironment towards a pro-chondrogenic scenario. Concerning T cells, miRNAs reported to reduce activation/proliferation resulted in a more abundant 2.41 ratio, mainly due to miR-24-3p and miR-21-5p within the >1% group (Table 2). miR-24-3p represses IFNγ [88], inhibits T cell proliferation and Th1/Th17 differentiation [89] and, by limiting IL4 production [90], also Th2 differentiation. miR-21-5p has several activities: it inhibits IFNγ and Th1 polarization [91,92] and positively regulates IL-10 secretion [93]. Intriguingly, IFNγ expression was described to be reduced by 5 EV-miRNAs (miR-24-3p/21-5p/125b/5p/29a-3p/27a-3p) [44]. Overall, EV-miRNAs are predicted to modulate T cell activation and proliferation and confirm published in vitro results [21]. Nevertheless, future studies might alter the overall weight and significance of EV-embedded miRNAs. As an example, even if not directly reported yet for miR-92a-3p, other miRNAs of its family cluster (miR-17-92) such as miR-17-5p and miR-19b-3p were reported to enhance T cell proliferation, Th1 differentiation and cytokine production with this phenotype abolished by miR-17-92 cluster deletion [94]. Eventually, concerning T reg, miR-21-5p tipped the balance in favor of their activation and proliferation (Table 2). miR-21-5p is upregulated in T reg and positively regulates Foxp3 and IL-10 expression indirectly [95,96]. Regarding miR-24-3p which is highly abundant and able to reverse the pro/con ratio, conflicting results were published. Its expression was reported to be both increased [97] and decreased [98] in T reg. Moreover, its overexpression was shown to induce T reg generation and differentiation in vitro [99]. Such differences might be due to lack of homogeneity in the experimental settings and future studies will be necessary. Overall, our miRNA data support the in vitro T reg expansion given by secretome of ASCs treated with OA-SF.

Eventually, our data showed that OA-SF has a profound effect on the most abundantly released soluble factors and EV-miRNAs, albeit that a straight forwarded direction for protection vs. destruction in the OA setting is of difficult definition. In fact, in the significant upregulation of both IGFPBs, the most expressed factors suggest a potential increase in the IGF1 reservoir at the cartilage level. For other categories the picture is less clear. For example, the appearance of IL17R and IL21R might indicate an increased binding of potentially harmful and inflammatory cytokines, although the reduction of IL6ST and TNFRSF1A could decrease the overall effect. Regarding ECM stability, depletion of TIMP2 and Serpine1 might weaken the secretome inhibitory effect on MMPs, as well as Follistatin reduction on proteoglycans destruction repression, although the general result could be buffered by Cathepsin S downregulation. The same duplicity is present also for PLAUR, which is highly increased after OA-SF, and has the dual role of being both a direct plasmin activator and an indirect chondrocyte proliferation stimulator. Regarding EV-miRNAs, the OA-SF effect is less pronounced, even if PCA and hierarchical clustering were able to sharply discriminate treated vs. untreated samples. Of note, gained/upregulated miRNAs resulted in preferentially targeting molecules involved in ECM and chondrocyte/cartilage development, rather than macrophage homeostasis. As with secreted factors, the overall scenario is tricky. In fact, upregulated miRNAs that are shared between EVs and OA cartilage target several receptors involved in proper cartilage homeostasis, such as TGFB, FGF and PDGF-AA. TGFB signaling plays a critical role during OA development with receptor deletion leading to cartilage degradation, hypertrophic chondrocyte and osteophyte formation [100]. Similarly, hypertrophy of chondrocytes is accompanied by the disappearance of FGFR3 expression since FGFR3 exerts a protective effect in OA by activating the RAS–MAPK and PI3K–AKT pathways [101]. Moreover, it was reported that PDGF-AA increased proteoglycan production in chondrocytes and promoted cartilage repair [102], suggesting that reduction in its receptor levels might promote OA. On the contrary, chondro-protective miR-193b-3p and miR92a-3p, both in the group with genetic weight > 1%, resulted in the upregulated of a > 3.5 factor. Due to their similar role in promoting *ACAN*, *COMP*, *COL2A1* and *SOX9* expression and aggrecan catabolism reduction, an eventual cartilage protection from proteolytic ECM destruction might be postulated. Intriguingly, downregulated miRNAs did not define sharp categories related to cartilage homeostasis, as well as both gained/upregulated and downregulated miRNAs for macrophages activity. Only T cell activation targeting might be reduced after OA-SF interaction due to increase of inducer miR-214-3p and decrease of preventer miR-125b-5p.

The main limitation of the study is the limited number of soluble factors and miRNAs scored. We are aware that many other players might be involved in the fine tuning regulation given by the secretome. Especially for miRNAs, the constantly increased knowledge in both their number and function will add new pieces of information to the herein-presented picture. Our data are the first attempt to give a molecular explanation to the ASCs effect in the OA environment, and for this reason we scored soluble factors and miRNAs with a solid description of their roles, in order to lay the foundation for future studies aimed at fine tuning the overall message.

In conclusion, soluble factors and EV-miRNAs released by ASCs after OA-SF treatment give a molecular explanation for both the in vitro results, showing effects of ASCs on immune cells and chondrocytes, and the efficacy of adipose-based therapies in the conservative treatment of the early phase of OA patients, especially in terms of pain reduction and cartilage homeostasis. The herein-presented data also support the hypothesis that ASCs are able to recruit T cells and monocyte/macrophages and, when in proximity, to impair their proliferation, revert their activation state and alter their catabolic cytokine production. In this frame, the two-way interaction between ASCs and the OA joint environment should lead to consider on adipose-derived cell-based therapies more suitable and effective than cell-free products obtained with or without chemical stimulation.

## Figures and Tables

**Table 1:** Cytokines from OA-SF. Values are presented as mean ± SD (*n* = 6). nd = not detected.

Analyte (pg/mL)											
IL-1α	IL-1β	IL-2	IL-4	IL-6	IL-8	IL-10	IL-12	IL-17A	IFNγ	TNFα	GM-CSF
2 ± 1	7 ± 1	13 ± 2	nd	865 ± 275	325 ± 74	12 ± 2	5 ± 1	nd	1 ± 1	48 ± 2	7 ± 2

**Table 2:** EV-miRNAs involved in OA-related cell types and mechanisms.

EV-miRNA	% EV-Weight	Cartilage Protection	M2 vs. M1 Macrophage	T Cell Activation	T Reg Activation
miR-193b-3p	2.79	+			
miR-24-3p	1.80	+	+	−	+/−
miR-21-5p	1.52	−		−	+
miR-92a-3p	1.24	+			
miR-125b-5p	0.92	+/−		−	
miR-221-3p	0.75	+/−		+	
miR-214-3p	0.67			+	
miR-222-3p	0.51	+	+		
miR-145-5p	0.43	+/−	−		−
miR-320a-3p	0.42	+			
miR-19b-3p	0.25	−		+	
miR-199a-3p	0.23	+			
miR-16-5p	0.20	−		−	
miR-34a-5p	0.20	−	+		
miR-130a-3p	0.13	+	−		
miR-30b-5p	0.13	−			
miR-29a-3p	0.13	+		−	+
miR-17-5p	0.12	+		+	
miR-106a-5p	0.11			+	
miR-27a-3p	0.08	+		−	−
let-7b-5p	0.07		+	+	
miR-138-5p	0.07	−			
miR-218-5p	0.07				−
miR-149-5p	0.07	+			
miR-27b-3p	0.05	+	−	−	−
miR-130b-3p	0.05			−	
miR-152-3p	0.05	+			
miR-210-3p	0.04	+		−	

**Table 3:** EV-miRNAs involved in OA-related cell types and mechanisms.

EV-miRNA	+/− OA-SF	Cartilage Protection	M2 vs. M1 Macrophage	T Cell Activation	T Reg Activation
miR-193b-3p	3.73	+			
miR-92a-3p	3.95	+			
miR-125b-5p	0.40	+/−		−	
miR-214-3p	3.82			+	
miR-30b-5p	0.37	−			
let-7b-5p	G		+	+	
miR-130b-3p	3.55			−	

## Data Availability

Data are contained within the article or Supplementary Materials.

## Supplementary Materials

The following are available online at https://www.mdpi.com/article/10.3390/cells10051072/s1, Figure S1: characterization of ASCs marker expression profile. Representative dot plots of mesenchymal and hemato-endothelial markers in ASCs, without (control condition, CM, green) and with (48 h supplementation, CM+OA-SF, blue) OA-SF in the growth medium. One representative cell isolate is shown, Figure S2: setting up the EV-dedicated flow cytometer. Four fluorescent populations (160, 200, 240, and 500 nm) were resolved from the instrument noise, Table S1: soluble factors released from the 3 ASCs under study after OA-SF treatment expressed as pg per million ASCs, Table S2: Biological Processes associated with identified secreted factors, Table S3: miRNAs identified in EVs, Table S4: EV-miRNAs falling in the first quartile of expression within SD ≤ 2 group, Table S5: first quartile miRNA-mRNA interactions defined by strong experimental evidence, Table S6: first quartile EV-miRNAs univocal targets, Table S7: genes shared between univocal targets of first quartile EV-miRNAs and first quartile of expression of OA patients cartilage transcripts, Table S8: enriched Biological Processes in Table S7 genes, Table S9: genes in Table S7 involved in either cartilage/chondrocyte development or extracellular matrix organization, Table S10: genes shared between univocal targets of first quartile EV-miRNAs and first quartile of expression of OA patients macrophage transcripts, Table S11: enriched Biological Processes in Table S10 genes, Table S12: genes in Table S10 involved in either macrophage differentiation or macrophage activation, Table S13: soluble factors released from ASCs after OA-SF treatment (ASC-SF) or under standard medium culture (ASC-CM) falling within the > 1 ng per million cells group in OA-SF treated ASCs, Table S14: EV-miRNAs released from ASCs after OA-SF treatment (ASC-SF) or under standard medium culture (ASC-CM) with C_RT_ falling within first quartile group in OA-SF treated ASCs, Table S15: genes targeted by gained/upregulated or downregulated miRNAs after OA-SF treatment and cleared from shared molecules between the two groups and filtered through either first quartile of expression of OA patients cartilage transcripts or first quartile of expression of OA patients macrophage transcripts, Table S16: genes in Table S15 involved in either cartilage/chondrocyte development or extracellular matrix organization or involved in either macrophage differentiation or macrophage activation.

## Abbreviations

ASCs	Adipose-derived mesenchymal Stem Cells
EVs	Extracellular Vesicles
miRNA	microRNA
T cell	T lymphocyte
T reg	Regulatory T lymphocyte
ECM	Extra Cellular Matrix
MFI	Mean Fluorescence Intensity
GO	Gene Ontology
OA	Osteo Arthritis
SF	Synovial Fluid
