# Supplementary material for: Cartilage Protective and Immunomodulatory Features of Osteoarthritis Synovial Fluid-Treated Adipose-Derived Mesenchymal Stem Cells Secreted Factors and Extracellular Vesicles-Embedded miRNAs
Source: Cells. 2021 Apr 30;10(5):1072. doi: 10.3390/cells10051072 (PMC8147187; doi:10.3390/cells10051072)
Supplement: Supplementary file 1 [file cells-10-01072-s001.zip › Supplementary Table S13_Cells.pdf]

Protein	ASC1-SF	ASC2-SF	ASC3-SF	ASC1-CM	ASC2-CM	ASC3-CM	RATIO SF *vs* CM	SD	P-VALUE
IGFBP4	1631395	2143627	2554479	175050	171227	140493	**13.34**	**4.49**	**0.0414**
IGFBP6	265160	286556	375787	86903	80689	88036	**3.62**	**0.61**	**0.0177**
SPP1	9487	102203	8371	635	720	871	55.50	74.91	0.3347
FST	37474	37025	12549	760241	572521	230230	**0.06**	**0.01**	**0.0001**
PF4	10216	14262	43639	nd	nd	nd	**G**	**-**	**-**
TIMP2	16928	17204	25151	185649	178783	212782	**0.10**	**0.01**	**0.0001**
SERPINE1	17677	23588	14280	98172	101532	96698	**0.19**	**0.04**	**0.0009**
PLAUR	16998	18777	19642	1145	2479	1759	**11.20**	**3.64**	**0.0399**
TIMP1	14615	14292	19164	31296	26876	32273	0.53	0.06	0.0060
IL6ST	9040	14278	6071	31798	67477	37234	**0.22**	**0.06**	**0.0020**
CD14	11552	11652	3712	899	1590	887	8.12	4.39	0.1067
TNFRSF1A	6231	4665	6755	11455	21271	18277	**0.38**	**0.16**	**0.0219**
DKK1	8351	6117	1408	11377	12392	8294	0.47	0.28	0.0822
VCAM1	3147	8895	4172	nd	nd	nd	**G**	**-**	**-**
MIF	4110	5841	5325	524	379	604	10.69	4.12	0.0552
MICB	4396	1810	7998	3046	1372	166	16.98	27.02	0.4134
CTSS	6374	3470	2128	20251	13506	10799	**0.26**	**0.06**	**0.0021**
IL17R	3480	2783	5453	nd	nd	nd	**G**	**-**	**-**
ANG	3388	3119	3197	3812	7500	6337	0.60	0.25	0.1140
SELL	2102	2881	2310	nd	nd	nd	**G**	**-**	**-**
VEGFA	1506	976	957	1561	1017	1376	0.87	0.15	0.4732
IL21R	1039	1971	233	nd	nd	nd	**G**	**-**	**-**
GDF15	1023	1069	912	4021	4772	4511	**0.23**	**0.03**	**0.0004**

nd: not detected; G: gained; SD: standard deviation. In bold, significant differential amount (Gained or Ratio > 2 or < 0.5 and p-value < 0.05).
