# Supplementary material for: Cartilage Protective and Immunomodulatory Features of Osteoarthritis Synovial Fluid-Treated Adipose-Derived Mesenchymal Stem Cells Secreted Factors and Extracellular Vesicles-Embedded miRNAs
Source: Cells. 2021 Apr 30;10(5):1072. doi: 10.3390/cells10051072 (PMC8147187; doi:10.3390/cells10051072)
Supplement: Supplementary file 1 [file cells-10-01072-s001.zip › Supplementary Table S14_Cells.pdf]

miRNA	ASC1-SF	ASC2-SF	ASC3-SF	ASC1-CM	ASC2-CM	ASC3-CM	RATIO SF *vs* SM	SD	P-VALUE
miR-520e-3p	10.32	8.20	9.08	nd	nd	nd	**G**	**-**	**-**
miR-518f-3p	8.81	12.87	9.56	nd	nd	nd	**G**	**-**	**-**
miR-523-3p	12.14	10.76	13.53	nd	nd	nd	**G**	**-**	**-**
miR-193b-3p	13.13	13.01	13.53	15.00	15.09	15.27	**3.73**	**0.44**	**0.0085**
miR-24-3p	13.52	14.45	13.59	12.88	13.32	13.25	0.63	0.17	0.0637
miR-21-5p	13.19	14.31	14.83	14.42	14.98	15.31	1.78	0.51	0.1178
miR-92a-3p	14.59	14.00	14.59	16.44	16.21	16.44	**3.95**	**0.59**	**0.0131**
miR-125b-5p	14.23	15.17	15.08	13.03	13.61	13.86	**0.40**	**0.05**	**0.0023**
miR-221-3p	14.83	15.06	15.45	14.92	14.08	15.00	0.77	0.28	0.2908
miR-214-3p	14.98	15.42	15.45	16.98	17.51	17.14	**3.82**	**0.53**	**0.0116**
miR-222-3p	15.06	15.99	16.02	14.63	15.10	14.81	0.57	0.16	0.0432
miR-484	16.06	15.54	16.04	18.37	18.56	19.06	**7.04**	**1.80**	**0.0284**
miR-145-5p	16.53	16.45	14.84	16.02	17.07	15.99	1.48	0.76	0.3882
miR-320a-3p	16.07	15.55	16.21	18.20	18.58	18.47	5.76	2.06	0.0571
miR-302a-3p	14.65	17.45	16.33	nd	nd	nd	**G**	**-**	**-**
miR-191-5p	16.28	17.24	15.63	16.42	16.56	16.49	1.18	0.60	0.6551
miR-574-3p	16.33	16.18	16.67	17.11	17.48	17.48	1.98	0.42	0.0561
miR-19b-3p	16.13	16.88	17.08	16.95	16.67	17.20	1.24	0.47	0.4698
miR-663b	17.98	16.30	16.13	21.07	20.65	21.44	22.82	15.70	0.1378
miR-199a-3p	16.26	16.79	17.44	16.88	17.69	17.97	1.61	0.22	0.0407
miR-224-5p	16.40	17.17	17.18	17.66	18.05	17.93	1.97	0.38	0.0475
miR-16-5p	16.13	17.19	17.80	18.23	18.58	19.25	3.20	0.93	0.0547
miR-100-5p	16.09	17.60	17.46	15.16	15.87	15.98	**0.39**	**0.12**	**0.0127**
miR-34a-5p	16.42	16.84	17.91	17.31	18.09	18.53	1.92	0.43	0.0657
miR-99a-5p	16.15	18.12	17.39	15.04	15.91	15.84	**0.34**	**0.12**	**0.0108**
miR-197-3p	17.67	17.06	17.66	18.00	18.55	18.71	2.04	0.78	0.1472
miR-20a-5p	16.77	17.52	18.27	16.86	16.42	17.25	0.67	0.34	0.2348
miR-328-3p	17.51	17.03	18.11	17.69	18.12	18.36	1.48	0.55	0.2698
miR-409-3p	18.19	17.04	17.50	19.34	20.74	20.18	7.19	5.41	0.1860
miR-130a-3p	17.12	17.70	18.01	17.84	17.91	18.56	1.42	0.25	0.1006
miR-132-3p	17.02	18.16	17.74	18.13	18.75	18.51	1.79	0.33	0.0535
miR-30b-5p	17.21	18.41	17.39	15.81	16.16	16.49	**0.37**	**0.16**	**0.0208**
miR-29a-3p	16.98	18.64	17.47	17.37	17.37	17.54	0.93	0.46	0.8168
miR-17-5p	17.14	18.21	17.84	18.69	18.06	18.51	1.81	1.03	0.3063
miR-106a-5p	17.20	18.24	18.07	19.02	18.05	18.83	2.03	1.35	0.3173
miR-30c-5p	17.62	18.61	17.45	15.40	15.81	16.21	**0.26**	**0.15**	**0.0134**
miR-382-5p	17.75	18.56	17.55	nd	nd	nd	**G**	**-**	**-**
miR-27a-3p	17.81	18.26	18.72	16.87	17.46	17.95	0.56	0.03	0.0015
miR-365a-3p	17.84	17.92	19.27	19.82	19.84	19.84	3.06	1.37	0.1212
miR-193a-5p	18.06	18.22	19.14	19.80	19.82	19.59	2.58	1.07	0.1249
let-7b-5p	18.62	18.71	18.29	nd	nd	nd	**G**	**-**	**-**
miR-138-5p	17.33	19.33	19.01	19.75	20.07	20.47	3.25	1.88	0.1739
miR-218-5p	17.92	18.66	19.16	17.56	17.11	17.73	0.50	0.24	0.0690
miR-149-5p	19.05	18.39	18.40	19.94	20.44	20.27	3.22	1.20	0.0851
miR-376a-3p	18.24	19.50	18.26	19.05	19.09	19.61	1.68	0.90	0.3208
miR-22-3p	18.95	20.13	16.96	19.76	19.98	19.76	3.20	3.28	0.3652
miR-99b-5p	18.53	19.17	19.34	16.09	17.08	16.96	**0.20**	**0.03**	**0.0005**
miR-27b-3p	18.82	19.10	19.27	18.16	18.88	19.51	0.89	0.27	0.5535
miR-130b-3p	18.82	19.11	19.50	20.84	20.71	21.35	**3.55**	**0.51**	**0.0131**
miR-152-3p	18.36	19.33	19.75	17.87	18.36	18.44	0.54	0.16	0.0380
miR-532-5p	18.64	19.76	19.41	20.91	20.92	21.11	3.44	1.31	0.0841
miR-210-3p	19.26	20.42	18.13	19.82	19.80	19.28	1.45	0.79	0.4278

nd: not detected; G: gained; SD: standard deviation. In bold. significant differential amount (Gained or Ratio > 2 or < 0.5 and p-value < 0.05).
