# Supplementary material for: Cartilage Protective and Immunomodulatory Features of Osteoarthritis Synovial Fluid-Treated Adipose-Derived Mesenchymal Stem Cells Secreted Factors and Extracellular Vesicles-Embedded miRNAs
Source: Cells. 2021 Apr 30;10(5):1072. doi: 10.3390/cells10051072 (PMC8147187; doi:10.3390/cells10051072)
Supplement: Supplementary file 1 [file cells-10-01072-s001.zip › Supplementary Table S1_Cells.pdf]

Protein	ASC1	ASC2	ASC3	Mean	SD	Description
IGFBP4	1631395	2143627	2554479	2109834	462469	Insulin-like growth factor-binding protein 4
IGFBP6	265160	286556	375787	309168	58678	Insulin-like growth factor-binding protein 6
SPP1	9487	102203	8371	40020	53855	Osteopontin (Bone sialoprotein 1)
FST	37474	37025	12549	29016	14262	Follistatin (Activin-binding protein)
PF4	10216	14262	43639	22706	18241	Platelet factor 4
TIMP2	16928	17204	25151	19761	4670	Metalloproteinase inhibitor 2
SERPINE1	17677	23588	14280	18515	4711	Plasminogen activator inhibitor 1
PLAUR	16998	18777	19642	18472	1348	Urokinase plasminogen activator surface receptor
TIMP1	14615	14292	19164	16024	2725	Metalloproteinase inhibitor 1
IL6ST	9040	14278	6071	9797	4155	Interleukin-6 receptor subunit beta
CD14	11552	11652	3712	8972	4555	Monocyte differentiation antigen CD14
TNFRSF1A	6231	4665	6755	5884	1087	Tumor necrosis factor receptor superfamily member 1A
DKK1	8351	6117	1408	5292	3545	Dickkopf-related protein 1
VCAM1	3147	8895	4172	5405	3066	Vascular cell adhesion protein 1
MIF	4110	5841	5325	5092	889	Macrophage migration inhibitory factor
MICB	4396	1810	7998	4735	3108	MHC class I polypeptide-related sequence B
CTSS	6374	3470	2128	3991	2170	Cathepsin S
IL17R	3480	2783	5453	3905	1385	Interleukin-17 receptor A
ANG	3388	3119	3197	3235	139	Angiogenin
SELL	2102	2881	2310	2431	404	L-selectin
VEGFA	1506	976	957	1147	312	Vascular endothelial growth factor A
IL21R	1039	1971	233	1081	870	Interleukin-21 receptor
GDF15	1023	1069	912	1001	81	Growth/differentiation factor 15
TNFRSF14	1011	485	1088	861	328	Tumor necrosis factor receptor superfamily member 14
CNTN2	248	829	1502	860	628	Contactin-2
PECAM1	454	550	1346	783	489	Platelet endothelial cell adhesion molecule
PDGFRB	78	1628	437	715	811	Platelet-derived growth factor receptor beta
ALCAM	439	535	1252	742	445	CD166 antigen
TYRO3	567	215	1318	700	564	Tyrosine-protein kinase receptor
LYVE1	763	816	81	553	410	Lymphatic vessel endothelial hyaluronic acid receptor 1
IL6	253	211	939	468	409	Interleukin-6
MICA	422	391	475	429	43	MHC class I polypeptide-related sequence A
CCL2	344	419	383	382	38	C-C motif chemokine 2
ICAM1	401	434	222	352	114	Intercellular adhesion molecule 1
FAS	551	263	233	349	176	Tumor necrosis factor receptor superfamily member 6
TNFRSF1B	523	398	58	327	241	Tumor necrosis factor receptor superfamily member 1B
IFNL2	160	351	507	339	174	Interferon lambda-2
TNFRSF17	330	245	394	323	75	Tumor necrosis factor receptor superfamily member 17
SCARB2	352	263	328	314	46	Lysosome membrane protein 2
TNFRSF10C	177	314	475	322	149	Tumor necrosis factor receptor superfamily member 10C
TNFRSF9	145	253	184	194	54	Tumor necrosis factor receptor superfamily member 9
LCN2	246	222	53	174	105	Neutrophil gelatinase-associated lipocalin
CXCL12	225	179	81	162	73	Stromal cell-derived factor 1
CCL18	197	175	105	159	48	C-C motif chemokine 18
MOK	132	167	179	159	25	MAPK/MAK/MRK overlapping kinase
IL10RB	131	171	111	138	31	Interleukin-10 receptor subunit beta
TNFRSF21	158	103	117	126	28	Tumor necrosis factor receptor superfamily member 21
SELE	162	64	118	115	49	E-selectin
NRG1	105	121	101	109	11	Pro-neuregulin-1
HAVCR1	56	121	148	108	47	Hepatitis A virus cellular receptor 1
ERBB3	188	49	60	99	77	Receptor tyrosine-protein kinase erbB-3
CD40LG	105	66	82	84	20	CD40 ligand (Tumor necrosis factor ligand superfamily member 5)
ENG	65	22	137	75	58	Endoglin
CXCL8	56	51	87	64	20	Interleukin-8

FLT3LG	70	50	28	49	21	Fms-related tyrosine kinase 3 ligand
CCL4	17	17	86	40	40	C-C motif chemokine 4
CCL5	55	23	8	29	24	C-C motif chemokine 5
IL1R1	24	3	72	33	35	Interleukin-1 receptor type 1
