# Supplementary material for: Cartilage Protective and Immunomodulatory Features of Osteoarthritis Synovial Fluid-Treated Adipose-Derived Mesenchymal Stem Cells Secreted Factors and Extracellular Vesicles-Embedded miRNAs
Source: Cells. 2021 Apr 30;10(5):1072. doi: 10.3390/cells10051072 (PMC8147187; doi:10.3390/cells10051072)
Supplement: Supplementary file 1 [file cells-10-01072-s001.zip › Supplementary Table S2_Cells.pdf]

| GO-term | Description | Factors |
| --- | --- | --- |
| GO:0030198 | extracellular matrix organization | TIMP1,SERPINE1,TIMP2,ICAM1,VCAM1,CTSS,ENG,SPP1,PECAM1 |
| GO:0006955 | Immune response | TNFRSF17,TNFRSF1A,MIF,CCL2,SELL,MICB,TIMP2,ICAM1,IL10RB,VCAM1,PF4,TNFRSF21,CD14,ALCAM,CXCL8,IFNL2,ANG,PLAUR,TNFRSF14,FAS,TNFRSF10C,CTSS,CD40LG,LCN2,TNFRSF1B,CXCL12,IL6,IL1R1,MICA,PECAM1,CCL5,TNFRSF9,CCL18,CCL4 |
| GO:0006954 | Inflammatory response | TNFRSF1A,MIF,TIMP1,CCL2,ICAM1,SCARB2,IGFBP4,IL10RB,VCAM1,PF4,TNFRSF21,CD14,CXCL8,SELE,TNFRSF14,FAS,TNFRSF10C,CD40LG,TNFRSF1B,SPP1,IL6,IL1R1,CCL5,TNFRSF9,CCL18,CCL4 |
| GO:0016477 | Cell migration | MIF,CCL2,SELL,PDGFRB,TYRO3,ICAM1,VCAM1,PF4,CXCL8,IL17RA,CNTN2,SELE,ANG,ENG,CXCL12,NRG1,IL6,PECAM1,CCL5,VEGFA,CCL18,CCL4 |
| GO:0060326 | Cell chemotaxis | CCL2,PDGFRB,VCAM1,PF4,CXCL8,IL17RA,ENG,CXCL12,IL6,CCL5,VEGFA,CCL18,CCL4 |
| GO:0050900 | Leukocyte migration | MIF,CCL2,SELL,ICAM1,VCAM1,PF4,CXCL8,IL17RA,SELE,CXCL12,IL6,PECAM1,CCL5,VEGFA,CCL18,CCL4 |
| GO:0030595 | Leukocyte chemotaxis | CCL2,PF4,CXCL8,IL17RA,CXCL12,IL6,CCL5,VEGFA,CCL18,CCL4 |
| GO:0072678 | T cell migration | CCL2,ICAM1,CXCL12 |
| GO:0002548 | Monocyte chemotaxis | CCL2,IL6,CCL5,CCL18,CCL4 |
| GO:0048246 | Macrophage chemotaxis | CCL2,CCL5 |
| GO:2000406 | Positive regulation of T cell migration | CXCL12,CCL5 |
| GO:0090026 | Positive regulation of monocyte chemotaxis | SERPINE1,CXCL12,CCL5 |
| GO:0072683 | T cell extravasation | CCL2,ICAM1 |
| GO:0097530 | Granulocyte migration | CCL2,CXCL8,IL17RA,PECAM1,CCL5,VEGFA,CCL18,CCL4 |
| GO:0071621 | Granulocyte chemotaxis | CCL2,CXCL8,IL17RA,CCL5,VEGFA,CCL18,CCL4 |
| GO:1990266 | Neutrophil migration | CCL2,CXCL8,PECAM1,CCL5,CCL18,CCL4 |
| GO:0030593 | Neutrophil chemotaxis | CCL2,CXCL8,CCL5,CCL18,CCL4 |
| GO:1902624 | Positive regulation of neutrophil migration | PF4,CXCL8,IL1R1 |
| GO:0090023 | Positive regulation of neutrophil chemotaxis | PF4,CXCL8 |
| GO:0002443 | Leukocyte mediated immunity | MIF,SELL,MICB,TIMP2,ICAM1,CD14,PLAUR,CTSS,CD40LG,LCN2,TNFRSF1B,IL6,MICA,PECAM1 |
| GO:0002446 | Neutrophil mediated immunity | MIF,SELL,TIMP2,CD14,PLAUR,CTSS,LCN2,TNFRSF1B,IL6,PECAM1 |
| GO:0002456 | T cell mediated immunity | MICB,ICAM1,MICA |
| GO:0001817 | Regulation of cytokine production | MIF,SERPINE1,PF4,TNFRSF21,CD14,IL17RA,CD40LG,IL6ST,IL6,IL1R1,TNFRSF9 |
| GO:0071356 | Cellular response to tumor necrosis factor | TNFRSF17,TNFRSF1A,CCL2,ICAM1,VCAM1,TNFRSF21,CXCL8,TNFRSF14,FAS,TNFRSF10C,CD40LG,TNFRSF1B,CCL5,TNFRSF9,CCL18,CCL4 |
| GO:0071347 | Cellular response to interleukin-1 | CCL2,ICAM1,CXCL8,IL1R1,CCL5,CCL18,CCL4 |
| GO:0071346 | Cellular response to interferon-gamma | CCL2,ICAM1,VCAM1,CCL5,CCL18,CCL4 |
| GO:0071354 | Cellular response to interleukin-6 | ICAM1,IL6ST,IL6 |
| GO:0031994 | Insulin-like growth factor I binding | IGFBP4,IGFBP6 |
| GO:0031995 | Insulin-like growth factor II binding | IGFBP4,IGFBP6 |
