# Supplementary material for: Cartilage Protective and Immunomodulatory Features of Osteoarthritis Synovial Fluid-Treated Adipose-Derived Mesenchymal Stem Cells Secreted Factors and Extracellular Vesicles-Embedded miRNAs
Source: Cells. 2021 Apr 30;10(5):1072. doi: 10.3390/cells10051072 (PMC8147187; doi:10.3390/cells10051072)
Supplement: Supplementary file 1 [file cells-10-01072-s001.zip › Supplementary Table S3_Cells.pdf]

Target Name	ASC1 C_{RT}	ASC2 C_{RT}	ASC3 C_{RT}	ASCs Mean	ASCs SD
hsa-miR-520e-3p	10.32	8.20	9.08	9.20	1.06
hsa-miR-518f-3p	8.81	12.87	9.56	10.41	2.16
hsa-miR-523-3p	12.14	10.76	13.53	12.14	1.39
hsa-miR-636	21.53	10.01	7.46	13.00	7.50
hsa-miR-193b-3p	13.13	13.01	13.53	13.22	0.27
hsa-miR-367-3p	17.12	9.26	14.18	13.52	3.97
hsa-miR-646	13.75	10.90	15.99	13.55	2.55
hsa-miR-24-3p	13.52	14.45	13.59	13.86	0.52
hsa-miR-21-5p	13.19	14.31	14.83	14.11	0.84
hsa-miR-92a-3p	14.59	14.00	14.59	14.39	0.34
hsa-miR-302c-3p	16.29	11.23	16.39	14.63	2.95
hsa-miR-125b-5p	14.23	15.17	15.08	14.83	0.52
hsa-miR-221-3p	14.83	15.06	15.45	15.11	0.31
hsa-miR-214-3p	14.98	15.42	15.45	15.29	0.26
hsa-miR-551b-3p	10.57	9.60	25.86	15.34	9.12
hsa-miR-222-3p	15.06	15.99	16.02	15.69	0.54
hsa-miR-657	18.75	13.25	15.40	15.80	2.77
hsa-miR-484	16.06	15.54	16.04	15.88	0.29
hsa-miR-145-5p	16.53	16.45	14.84	15.94	0.96
hsa-miR-320a-3p	16.07	15.55	16.21	15.95	0.35
hsa-miR-302a-3p	14.65	17.45	16.33	16.14	1.41
hsa-miR-1267	10.60	13.96	24.49	16.35	7.25
hsa-miR-191-5p	16.28	17.24	15.63	16.38	0.81
hsa-miR-615-3p	19.69	18.76	10.71	16.39	4.94
hsa-miR-574-3p	16.33	16.18	16.67	16.39	0.25
hsa-miR-19b-3p	16.13	16.88	17.08	16.70	0.50
hsa-miR-663b	17.98	16.30	16.13	16.80	1.02
hsa-miR-199a-3p	16.26	16.79	17.44	16.83	0.59
hsa-miR-224-5p	16.40	17.17	17.18	16.92	0.45
hsa-miR-16-5p	16.13	17.19	17.80	17.04	0.84
hsa-miR-100-5p	16.09	17.60	17.46	17.05	0.84
hsa-miR-34a-5p	16.42	16.84	17.91	17.05	0.77
hsa-miR-99a-5p	16.15	18.12	17.39	17.22	1.00
hsa-miR-197-3p	17.67	17.06	17.66	17.46	0.35
hsa-miR-20a-5p	16.77	17.52	18.27	17.52	0.75
hsa-miR-328-3p	17.51	17.03	18.11	17.55	0.54
hsa-miR-1183	19.70	15.28	17.72	17.56	2.22
hsa-miR-409-3p	18.19	17.04	17.50	17.58	0.58
hsa-miR-130a-3p	17.12	17.70	18.01	17.61	0.45
hsa-miR-132-3p	17.02	18.16	17.74	17.64	0.57
hsa-miR-30b-5p	17.21	18.41	17.39	17.67	0.65
hsa-miR-29a-3p	16.98	18.64	17.47	17.69	0.85
hsa-miR-17-5p	17.14	18.21	17.84	17.73	0.55
hsa-miR-106a-5p	17.20	18.24	18.07	17.83	0.55
hsa-miR-30c-5p	17.62	18.61	17.45	17.89	0.63
hsa-miR-382-5p	17.75	18.56	17.55	17.95	0.54
hsa-miR-520c-3p	20.84	21.43	12.22	18.16	5.15
hsa-miR-27a-3p	17.81	18.26	18.72	18.26	0.46
hsa-miR-365a-3p	17.84	17.92	19.27	18.35	0.80
hsa-miR-193a-5p	18.06	18.22	19.14	18.47	0.59
hsa-let-7b-5p	18.62	18.71	18.29	18.54	0.22
hsa-miR-138-5p	17.33	19.33	19.01	18.56	1.07
hsa-miR-218-5p	17.92	18.66	19.16	18.58	0.63

hsa-miR-149-5p	19.05	18.39	18.40	18.61	0.38
hsa-miR-376a-3p	18.24	19.50	18.26	18.67	0.72
hsa-miR-22-3p	18.95	20.13	16.96	18.68	1.60
hsa-miR-99b-5p	18.53	19.17	19.34	19.01	0.43
hsa-miR-27b-3p	18.82	19.10	19.27	19.07	0.23
hsa-miR-130b-3p	18.82	19.11	19.50	19.15	0.34
hsa-miR-152-3p	18.36	19.33	19.75	19.15	0.71
hsa-miR-532-5p	18.64	19.76	19.41	19.27	0.58
hsa-miR-210-3p	19.26	20.42	18.13	19.27	1.15
hsa-miR-31-5p	18.89	20.05	18.97	19.30	0.65
hsa-miR-483-5	19.14	19.46	19.56	19.38	0.22
hsa-miR-346	20.57	19.73	17.97	19.42	1.33
hsa-miR-127-3p	18.92	20.11	19.31	19.44	0.61
hsa-miR-30a-5p	19.04	19.73	19.58	19.45	0.36
hsa-miR-23a-3p	18.77	20.06	19.70	19.51	0.67
hsa-miR-25-3p	18.95	19.89	19.71	19.52	0.50
hsa-miR-662	22.83	15.88	19.93	19.54	3.49
hsa-miR-423-5p	19.30	19.04	20.38	19.57	0.71
hsa-miR-106b-5p	19.02	20.42	19.30	19.58	0.74
hsa-let-7a-5p	20.32	19.05	19.61	19.66	0.64
hsa-miR-26a-5p	19.53	20.12	19.55	19.73	0.33
hsa-miR-342-3p	19.63	20.54	19.11	19.76	0.73
hsa-miR-886-3p	20.67	19.95	19.07	19.89	0.80
hsa-miR-1260a	21.61	19.61	18.49	19.90	1.58
hsa-miR-148a-3p	19.46	20.32	20.98	20.25	0.76
hsa-miR-29c-3p	19.10	21.10	20.56	20.25	1.04
hsa-miR-520d-3p	15.31	27.19	18.31	20.27	6.18
hsa-miR-361-5p	20.01	20.60	20.49	20.37	0.31
hsa-miR-660-5p	19.78	20.25	21.27	20.43	0.76
hsa-miR-661	22.11	19.88	19.31	20.43	1.48
hsa-miR-370-3p	20.64	20.00	20.70	20.45	0.39
hsa-miR-939-5p	20.95	19.40	21.10	20.48	0.94
hsa-miR-31-3p	20.38	20.68	20.43	20.50	0.16
hsa-let-7e-5p	21.17	19.81	20.55	20.51	0.68
hsa-miR-93-5p	19.81	20.59	21.18	20.53	0.69
hsa-miR-296-5p	20.77	20.33	20.54	20.55	0.22
hsa-miR-485-3p	19.25	21.25	21.31	20.60	1.17
hsa-miR-654-5p	21.17	20.35	20.54	20.69	0.43
hsa-miR-494-3p	20.60	21.20	20.62	20.81	0.34
hsa-miR-223-3p	22.23	19.29	21.04	20.86	1.48
hsa-miR-28-3p	20.00	21.26	21.31	20.86	0.74
hsa-miR-1233-3p	21.86	21.07	19.65	20.86	1.12
hsa-miR-143-3p	20.64	21.26	20.72	20.87	0.33
hsa-miR-518d-3p	15.27	22.17	25.70	21.04	5.30
hsa-miR-339-5p	21.61	20.08	21.56	21.08	0.87
hsa-miR-886-5p	21.10	22.14	20.18	21.14	0.98
hsa-miR-331-3p	21.07	21.28	21.22	21.19	0.11
hsa-miR-601	18.45	21.36	23.77	21.19	2.67
hsa-miR-212-3p	21.38	21.25	21.16	21.26	0.11
hsa-miR-195-5p	20.12	21.17	22.59	21.30	1.24
hsa-let-7c-5p	20.60	21.77	21.59	21.32	0.63
hsa-miR-146b-5p	21.09	22.25	21.01	21.45	0.69
hsa-miR-410-3p	21.19	21.78	21.42	21.46	0.30
hsa-miR-345-5p	21.11	22.22	21.16	21.50	0.63
hsa-miR-424-3p	22.09	21.57	20.90	21.52	0.60
hsa-miR-483-3p	21.55	20.53	22.54	21.54	1.01

hsa-miR-135b-3p	22.95	22.20	19.60	21.58	1.76
hsa-miR-766-3p	22.51	21.28	21.01	21.60	0.80
hsa-miR-26b-5p	21.63	21.59	21.59	21.60	0.02
hsa-miR-186-5p	21.03	22.22	21.60	21.62	0.59
hsa-miR-15b-5p	21.08	21.67	22.19	21.65	0.55
hsa-miR-103a-3p	21.72	21.89	21.57	21.72	0.16
hsa-miR-30d-3p	21.25	21.85	22.11	21.74	0.44
hsa-miR-10b-3p	21.78	21.93	21.76	21.82	0.09
hsa-miR-433-3p	22.33	20.65	22.61	21.86	1.06
hsa-miR-331-5p	24.77	20.86	20.13	21.92	2.49
hsa-miR-181a-5p	21.32	23.39	21.22	21.98	1.22
hsa-miR-129-2-3p	24.03	19.12	22.90	22.02	2.57
hsa-miR-638	22.89	21.86	21.32	22.03	0.80
hsa-miR-204-5p	21.04	22.78	22.65	22.15	0.97
hsa-miR-34a-3p	20.89	22.58	23.06	22.18	1.14
hsa-miR-301a-3p	22.79	21.15	22.59	22.18	0.89
hsa-miR-335-5p	22.65	22.55	21.36	22.19	0.72
hsa-miR-128-3p	21.96	22.22	22.40	22.19	0.22
hsa-miR-34b-3p	21.99	22.04	22.64	22.22	0.36
hsa-miR-532-3p	22.05	22.31	22.45	22.27	0.20
hsa-miR-134-5p	22.43	22.02	22.53	22.33	0.27
hsa-miR-1290	22.77	23.22	20.99	22.33	1.18
hsa-miR-95-3p	21.99	22.30	22.77	22.36	0.39
hsa-miR-19a-3p	21.25	22.51	23.33	22.37	1.05
hsa-miR-215-5p	23.47	22.59	21.25	22.44	1.12
hsa-miR-199b-5p	22.35	22.13	22.87	22.45	0.38
hsa-miR-648	23.66	21.56	22.17	22.46	1.08
hsa-miR-192-5p	22.83	21.84	22.83	22.50	0.57
hsa-miR-34c-5p	21.78	22.19	23.53	22.50	0.91
hsa-miR-7-1-3p	22.38	22.90	22.34	22.54	0.31
hsa-miR-30a-3p	22.86	22.47	22.37	22.57	0.26
hsa-miR-379-5p	22.88	21.37	23.46	22.57	1.08
hsa-miR-323a-3p	22.09	23.23	22.44	22.59	0.59
hsa-miR-659-3p	23.65	21.74	22.45	22.61	0.96
hsa-miR-30e-3p	23.05	22.60	22.31	22.65	0.37
hsa-miR-491-5p	24.13	19.75	24.13	22.67	2.53
hsa-let-7g-5p	22.07	23.46	22.54	22.69	0.70
hsa-miR-193b-5p	23.79	21.57	22.75	22.70	1.11
hsa-miR-320b	22.88	22.83	22.82	22.85	0.03
hsa-miR-874-3p	24.07	22.51	22.00	22.86	1.08
hsa-miR-324-5p	22.11	24.59	21.96	22.88	1.48
hsa-miR-324-3p	21.84	23.91	22.99	22.91	1.04
hsa-miR-150-5p	25.58	20.68	22.56	22.94	2.48
hsa-miR-1303	24.22	23.08	21.52	22.94	1.36
hsa-miR-486-5p	22.13	23.40	23.31	22.94	0.71
hsa-miR-650	22.91	22.33	23.67	22.97	0.68
hsa-miR-744-5p	22.48	22.98	23.58	23.01	0.55
hsa-miR-543	22.96	23.66	22.60	23.07	0.54
hsa-miR-10a-5p	20.99	24.70	23.57	23.09	1.90
hsa-miR-455-3p	23.22	23.63	22.64	23.16	0.50
hsa-miR-374a-5p	22.96	23.21	23.44	23.20	0.24
hsa-miR-625-3p	23.73	22.95	22.98	23.22	0.44
hsa-miR-199a-5p	22.72	22.45	24.85	23.34	1.32
hsa-miR-590-5p	22.13	25.06	22.97	23.39	1.51
hsa-miR-452-5p	23.03	23.73	23.55	23.44	0.36
hsa-miR-146a-5p	23.69	24.67	21.97	23.45	1.36

hsa-miR-487b-3p	23.17	23.59	23.59	23.45	0.24
hsa-miR-1291	23.39	24.20	22.88	23.49	0.66
hsa-let-7d-5p	24.22	22.98	23.55	23.58	0.62
hsa-miR-758-3p	25.42	22.22	23.35	23.66	1.62
hsa-miR-133a-3p	24.84	25.72	20.61	23.72	2.73
hsa-miR-29b-3p	23.47	23.83	23.91	23.74	0.24
hsa-miR-330-3p	23.89	24.35	23.43	23.89	0.46
hsa-miR-22-5p	23.35	24.22	24.15	23.90	0.48
hsa-miR-155-5p	23.07	24.43	24.35	23.95	0.76
hsa-miR-505-3p	24.09	23.62	24.15	23.95	0.29
hsa-miR-339-3p	22.53	24.25	25.20	23.99	1.35
hsa-miR-655-3p	25.08	26.30	20.64	24.00	2.98
hsa-miR-185-5p	23.06	24.87	24.11	24.01	0.91
hsa-miR-28-5p	23.26	24.45	24.45	24.05	0.69
hsa-miR-203a-3p	24.62	22.08	26.00	24.23	1.99
hsa-miR-671-3p	24.37	24.95	23.41	24.24	0.77
hsa-miR-140-5p	22.94	25.70	24.31	24.32	1.38
hsa-miR-337-5p	24.13	24.33	24.57	24.34	0.22
hsa-miR-572	26.03	23.56	23.74	24.44	1.38
hsa-miR-411-5p	22.93	25.25	25.58	24.59	1.45
hsa-miR-99b-3p	25.23	23.65	25.05	24.64	0.86
hsa-miR-455-5p	24.13	26.18	23.74	24.68	1.31
hsa-miR-548b-5p	25.43	24.01	24.66	24.70	0.71
hsa-miR-148b-3p	23.93	25.11	25.15	24.73	0.70
hsa-miR-151a-3p	23.35	26.09	24.76	24.74	1.37
hsa-miR-424-5p	23.99	26.43	23.83	24.75	1.46
hsa-miR-708-5p	24.29	26.01	24.04	24.78	1.07
hsa-miR-29a-5p	23.84	24.92	25.63	24.80	0.91
hsa-miR-214-5p	24.49	24.50	25.75	24.92	0.72
hsa-miR-502-3p	24.27	24.78	25.81	24.95	0.78
hsa-miR-15a-5p	24.42	25.00	25.46	24.96	0.52
hsa-miR-381-3p	25.46	24.52	25.05	25.01	0.47
hsa-miR-656-3p	25.49	24.30	25.25	25.01	0.63
hsa-miR-500a-5p	25.10	25.22	24.81	25.04	0.21
hsa-miR-645	26.45	24.91	23.78	25.05	1.34
hsa-miR-1227-3p	27.08	24.54	23.77	25.13	1.73
hsa-miR-432-3p	24.75	26.09	24.68	25.18	0.79
hsa-miR-125b-1-3p	24.88	24.78	25.92	25.19	0.63
hsa-miR-222-5p	26.55	23.81	25.32	25.23	1.37
hsa-miR-889-3p	24.25	26.49	25.05	25.26	1.14
hsa-miR-597-5p	26.45	23.89	25.72	25.35	1.32
hsa-miR-1255b-5p	26.92	24.65	24.62	25.40	1.32
hsa-miR-20b-5p	24.78	24.67	26.81	25.42	1.21
hsa-miR-142-3p	25.74	26.41	24.33	25.50	1.06
hsa-miR-942-5p	26.04	25.88	25.10	25.67	0.50
hsa-miR-1271-5p	25.57	25.95	25.59	25.70	0.21
hsa-miR-140-3p	24.59	26.02	26.70	25.77	1.08
hsa-miR-124-3p	26.60	25.89	24.89	25.79	0.86
hsa-miR-539-5p	24.79	26.55	26.06	25.80	0.91
hsa-miR-1285-3p	27.08	26.01	24.45	25.85	1.32
hsa-miR-136-3p	25.61	26.03	25.91	25.85	0.22
hsa-miR-425-3p	27.45	24.44	25.92	25.94	1.51
hsa-miR-629-3p	26.14	26.87	25.21	26.08	0.83
hsa-miR-493-3p	26.52	26.79	25.65	26.32	0.60
hsa-miR-190a-5p	27.58	27.15	24.46	26.40	1.69
hsa-miR-125a-5p	27.45	25.13	26.90	26.49	1.21

hsa-miR-616-5p	27.08	26.21	26.54	26.61	0.44
hsa-miR-301b-3p	27.83	26.64	25.56	26.68	1.14
