# Supplementary material for: Cartilage Protective and Immunomodulatory Features of Osteoarthritis Synovial Fluid-Treated Adipose-Derived Mesenchymal Stem Cells Secreted Factors and Extracellular Vesicles-Embedded miRNAs
Source: Cells. 2021 Apr 30;10(5):1072. doi: 10.3390/cells10051072 (PMC8147187; doi:10.3390/cells10051072)
Supplement: Supplementary file 1 [file cells-10-01072-s001.zip › Supplementary Table S4_Cells.pdf]

Target Name	Mean C_{RT}	SD	% whole EVs weight
miR-520e-3p	9.20	1.06	45.50
*miR-518f-3p**	*10.41*	*2.16*	*19.63*
miR-523-3p	12.14	1.39	5.91
miR-193b-3p	13.22	0.27	2.79
miR-24-3p	13.86	0.52	1.80
miR-21-5p	14.11	0.84	1.52
miR-92a-3p	14.39	0.34	1.24
miR-125b-5p	14.83	0.52	0.92
miR-221-3p	15.11	0.31	0.75
miR-214-3p	15.29	0.26	0.67
miR-222-3p	15.69	0.54	0.51
miR-484	15.88	0.29	0.44
miR-145-5p	15.94	0.96	0.43
miR-320a-3p	15.95	0.35	0.42
miR-302a-3p	16.14	1.41	0.37
miR-191-5p	16.38	0.81	0.31
miR-574-3p	16.39	0.25	0.31
miR-19b-3p	16.70	0.50	0.25
miR-663b	16.80	1.02	0.23
miR-199a-3p	16.83	0.59	0.23
miR-224-5p	16.92	0.45	0.22
miR-16-5p	17.04	0.84	0.20
miR-100-5p	17.05	0.84	0.20
miR-34a-5p	17.05	0.77	0.20
miR-99a-5p	17.22	1.00	0.18
miR-197-3p	17.46	0.35	0.15
miR-20a-5p	17.52	0.75	0.14
miR-328-3p	17.55	0.54	0.14
miR-409-3p	17.58	0.58	0.14
miR-130a-3p	17.61	0.45	0.13
miR-132-3p	17.64	0.57	0.13
miR-30b-5p	17.67	0.65	0.13
miR-29a-3p	17.69	0.85	0.13
miR-17-5p	17.73	0.55	0.12
miR-106a-5p	17.83	0.55	0.11
miR-30c-5p	17.89	0.63	0.11
miR-382-5p	17.95	0.54	0.11
miR-27a-3p	18.26	0.46	0.08
miR-365a-3p	18.35	0.80	0.08
miR-193a-5p	18.47	0.59	0.07
let-7b-5p	18.54	0.22	0.07
miR-138-5p	18.56	1.07	0.07
miR-218-5p	18.58	0.63	0.07
miR-149-5p	18.61	0.38	0.07
miR-376a-3p	18.67	0.72	0.06
miR-22-3p	18.68	1.60	0.06
miR-99b-5p	19.01	0.43	0.05
miR-27b-3p	19.07	0.23	0.05
miR-130b-3p	19.15	0.34	0.05
miR-152-3p	19.15	0.71	0.05
miR-532-5p	19.27	0.58	0.04
miR-210-3p	19.27	1.15	0.04

** Included even if not falling in the SD ≤ 2 group*
