# Supplementary figures and images for: Cartilage Protective and Immunomodulatory Features of Osteoarthritis Synovial Fluid-Treated Adipose-Derived Mesenchymal Stem Cells Secreted Factors and Extracellular Vesicles-Embedded miRNAs
Source: Cells. 2021 Apr 30;10(5):1072. doi: 10.3390/cells10051072 (PMC8147187; doi:10.3390/cells10051072)

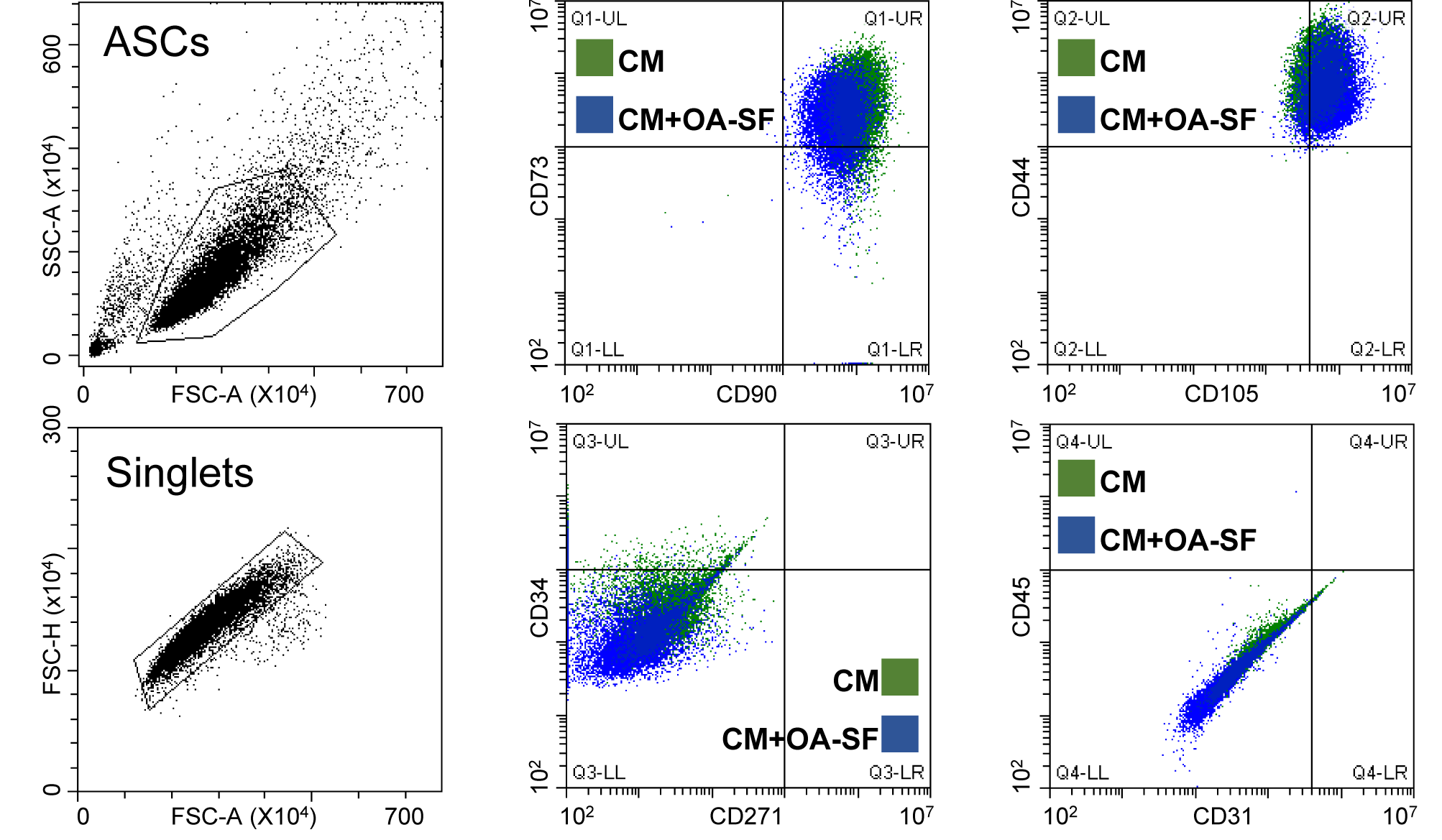

Supplement: Supplementary file 1 [file cells-10-01072-s001.zip › Supplementary Figure S1_Cells.tif]

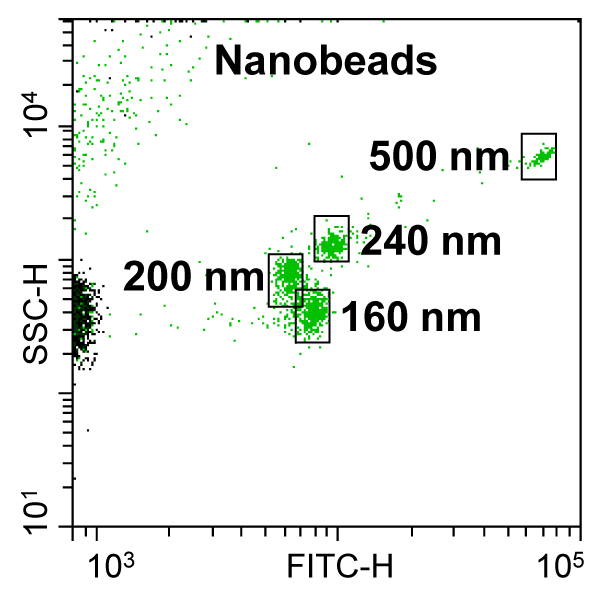

Supplement: Supplementary file 1 [file cells-10-01072-s001.zip › Supplementary Figure S2_Cells.tif]
